# Supplementary material for: Learning from electronic prescribing errors: a mixed methods study of junior doctors’ perceptions of training and individualised feedback data
Source: BMJ Open. 2022 Dec 21;12(12):e056221. doi: 10.1136/bmjopen-2021-056221 (PMC9772675; doi:10.1136/bmjopen-2021-056221)
Supplement: Supplementary data [file bmjopen-2021-056221supp001.pdf]

Appendix 1 Questionnaire template

Appendix 1:

Junior doctors' prescribing safety survey

Improving safety and effectiveness of electronic prescribing – how can training and learning from errors be improved?

Invitation: **All foundation year 1 and 2 doctors** at Imperial College Healthcare NHS Trust.

This questionnaire forms part of a study that aims to improve prescribing safety in hospitals with electronic prescribing systems. It centres on how the Trust can help make it easier to prescribe safely and effectively, particularly in the way in which electronic prescribing training and feedback about errors are currently provided. It also asks about your preferred methods for receiving feedback on your prescribing errors and learning from prescribing errors. Participation is voluntary, and all information will be treated confidentially. **If you have any questions, please contact monsey.mcleod@nhs.net on behalf of the Imperial Prescribing Feedback team.**

In the following questions, please select the answer that best represents your opinion by circling it.
(**SD**= Strongly Disagree, **D**= Disagree, **N**= Neutral, **A**= Agree, **SA**= Strongly Agree)

A. YOUR PRESCRIBING AND FEEDBACK EXPERIENCE

I am confident in prescribing the correct and appropriate **non-intravenous** medications for patients via Cerner.		SD	D	N	A	SA
I am confident in prescribing the correct and appropriate **intravenous** medications for patients via Cerner.		SD	D	N	A	SA
I would prefer to use a paper drug chart.	Never used	SD	D	N	A	SA
I think I am always informed if I make a prescribing error.		SD	D	N	A	SA
It concerns me I may make repeated prescribing errors because I am not informed of them initially.		SD	D	N	A	SA
I think receiving formal feedback on prescribing errors makes it less likely that I make errors in the future.		SD	D	N	A	SA
Any feedback I receive is generally unscheduled/unplanned.		SD	D	N	A	SA

B. QUALITY IMPROVEMENT

I believe electronic prescribing has reduced the number of prescribing errors that result in actual patient harm.	Don't know	SD	D	N	A	SA
I believe monitoring and learning from prescribing errors is important.		SD	D	N	A	SA
I believe prescribers should receive feedback about their own prescribing errors.		SD	D	N	A	SA
I believe being aware of my own prescribing errors is important to improve my prescribing.		SD	D	N	A	SA

C. TRAINING

Overall, I am satisfied with electronic prescribing training I receive.		SD	D	N	A	SA
I am satisfied with the following: ePrescribing training provided at induction.	Not received	SD	D	N	A	SA
I am satisfied with the ePrescribing classroom training session provided by the Trust Information Communications and Technology (ICT) team.	Not received	SD	D	N	A	SA
I am satisfied with the e-learning prescribing training module.	Not received	SD	D	N	A	SA
I am satisfied with the specialty-specific prescribing training I receive.	Not received	SD	D	N	A	SA

15

Appendix 1 Questionnaire template

I am satisfied with the prescribing training provided as part of the postgraduate education sessions for F2/CMT (covers general prescribing safety including electronic prescribing tips).	Not received	SD D N A SA

D. RESOURCES

Overall, I am satisfied with the resources I can access to prescribe safely.		SD D N A SA
When relevant, I like to use the 'EPA quick reference guide' and crib sheets on the Source to help me prescribe.	Not used	SD D N A SA
In general, I mostly contact the pharmacy EPA team for technical prescribing queries relating to the Cerner system.	Not used	SD D N A SA
In general, I mostly ask **nurses** for help whilst prescribing (for technical Cerner-specific queries or medication-related queries).	Not used	SD D N A SA
In general, I mostly ask the **ward pharmacist** for help whilst prescribing (for technical Cerner-specific queries or medication-related queries).	Not used	SD D N A SA
In general, I mostly ask **my peers** for help whilst prescribing (for technical Cerner-specific queries or medication-related queries).	Not used	SD D N A SA
In general, I mostly ask a **CMT** for help whilst prescribing (for technical Cerner-specific queries or medication-related queries).	Not used	SD D N A SA
In general, I mostly ask the **consultant** for help whilst prescribing (for technical Cerner-specific queries or medication-related queries).	Not used	SD D N A SA
In general, I mostly ask nurses for help whilst prescribing **out-of-hours**	Not used	SD D N A SA
In general, I mostly ask the on-call pharmacist for help whilst prescribing **out-of-hours**	Not used	SD D N A SA
In general, I mostly ask my peers for help whilst prescribing **out-of-hours**	Not used	SD D N A SA
In general, I mostly ask a CMT for help whilst prescribing **out-of-hours**	Not used	SD D N A SA

E. PROSPECTIVE FEEDBACK (specific error) INCLUDING EXAMPLE SCENARIOS

I want to know about all **major** prescribing errors I make.	SD D N A SA
I want to know about **all** prescribing errors I make, however minor.	SD D N A SA
I **ONLY** want to know about prescribing errors I make that have the potential to cause patient harm.	SD D N A SA
I would like verbal feedback to be provided to me at the time the error is detected.	SD D N A SA
I would like to be able to receive an instant message when a prescribing error I've made has been detected.	SD D N A SA
I would like to be able to receive an email within the Cerner message centre when a prescribing error I've made has been detected.	SD D N A SA

I would like to receive feedback if I made this type of prescribing error:

An 82-year-old male patient with hospital acquired pneumonia was prescribed temocillin 2g IV BD and amoxicillin IV 1g TDS. He had a penicillin allergy with known anaphylaxis but his allergy status had not been completed on the electronic prescribing system. The error was detected and corrected before it reached the patient.	SD D N A SA
A 67-year-old female admitted for elective right hip replacement was started on prophylactic antibiotics at induction. Cefuroxime 1.5g IV TDS was prescribed post-op, intended for 24 hours but no stop date was prescribed. The patient received cefuroxime 1.5g IV TDS for two extra days before it was stopped. The patient has not reported any side effects from the prolonged course.	SD D N A SA
A 55-year-old male with type 2 diabetes and hypertension was admitted. One of their medications, ramipril 10mg OD, was not prescribed and the patient missed their dose for 3 days over the weekend. Their blood pressure had increased gradually and is now 190/110 mmHg.	SD D N A SA

Appendix 1 Questionnaire template

A 11-month old girl was prescribed amoxicillin 125mg/5ml suspension twice. One was for 2.5ml TDS for one week, and the other for 5ml TDS for one week. The error was corrected before it reached the patient.	SD	D	N	A	SA

F. RETROSPECTIVE FEEDBACK (error trends specific to my own prescribing)

I would like to receive a regular summary of my prescribing errors.	SD	D	N	A	SA
I would like to have a way of tracking prescribing errors I make over time.	SD	D	N	A	SA
I would like the opportunity to regularly review my prescribing errors with **my peers**.	SD	D	N	A	SA
I would like the opportunity to regularly review my prescribing errors with a **pharmacist**.	SD	D	N	A	SA
I would like the opportunity to regularly review my prescribing errors with a **consultant**.	SD	D	N	A	SA
Feedback (review) sessions about prescribing errors of relevance to my specialty would be most valuable on the **ward**, (e.g. at handover, as part of ward round).	SD	D	N	A	SA

G. AGGREGATED RETROSPECTIVE FEEDBACK (of prescribing errors within Imperial)

I would like to know about other common prescribing errors besides my own.	SD	D	N	A	SA
I would like more information about common prescribing errors in existing training sessions.	SD	D	N	A	SA
I would like to learn about prescribing errors and how to prevent them in new dedicated prescribing improvement sessions.	SD	D	N	A	SA
I would like to receive a regular summary of **common** prescribing errors within my specialty.	SD	D	N	A	SA
I would like to receive a regular summary of **rare but serious** prescribing errors within my **specialty**.	SD	D	N	A	SA
I would like to receive a regular summary of **rare but serious** prescribing errors within the **trust**.	SD	D	N	A	SA

Please answer the following questions.
If you had enough time, what three things would make it easier for you to prescribe safely and efficiently?

What are the main concerns regarding the current feedback you receive, if any, on prescribing errors?

Sex: F / M	Grade:	Medical school qualified from:

Please email monsey.mcleod@nhs.net on behalf of the Imperial Prescribing Feedback team for any of the following:

- ☐ I would like to receive a copy of the survey results
- ☐ I am happy to be contacted to clarify my answers above
- ☐ I am happy to be contacted about future improvement projects

Thank you for completing this questionnaire.
Please return to this to the Postgraduate Medical Education team at your site (directly or internal mail).

17
