# Supplementary material for: Learning from electronic prescribing errors: a mixed methods study of junior doctors’ perceptions of training and individualised feedback data
Source: BMJ Open. 2022 Dec 21;12(12):e056221. doi: 10.1136/bmjopen-2021-056221 (PMC9772675; doi:10.1136/bmjopen-2021-056221)

Appendix 2 Interview Topic Guide

Prescribing Safety Feedback Interview

Thank you for taking the time to participate this study. My name is XX and I am currently doing some research into feedback on prescribing errors within this trust.
Have you had a chance to look at the participant information sheet? Do you still have any questions? Are you happy for us to take notes? Are you happy to sign this consent form? No identifiable information is being recorded.

This interview is part of a larger study to explore the views of junior doctors and other stakeholders about providing feedback concerning prescribing errors. The interview focuses on how best to provide feedback to foundation year doctors to help make it easier to prescribe safely on the electronic system. This includes receiving feedback on prescribing errors as well as tips for how to prevent them. Participation is voluntary, and these interviews are completely anonymous.

Selection of prescribing error related feedback strategies

We know from a recent survey at this trust that FY1 and FY2 doctors do want to receive feedback about their own prescribing errors and also learn from errors elsewhere. In response to this, we are now looking to get your views on a range of different methods for receiving prescribing feedback.

PART 1

I will show you a few ways of providing feedback to support learning from prescribing errors

and would like your feedback on each strategy. [ACTION: give out documents – see below].

Questions to ask while they are looking at the documents:

1. Which of these feedback reports do you prefer? Please rank in order of preference.

- *[Wait until they have put them in order of preference, indicating which they think is*

best to worst (or if the same)]

2. Can you tell me the reasons for your answer?

3. What changes to this feedback document would make this more useful to you?

a. Prompts: What would you like to see changed about the feedback concerning

prescribing errors?

b. Prompts: Do you identify any problems with this feedback report? If you do,

which problems?

c. Prompts: What do you like about the presentation style?

74

d. Prompts: Is the information presented clear to you? Is the text big enough?

Appendix 2 Interview Topic Guide

Do you like the length of the feedback document or do you think we need to add/remove some information?

4. If you want to receive feedback, how often do you want to receive a document like these?

a. Prompts: Would you prefer to receive a feedback document weekly or monthly or other?

PART 2

Thank you, now I would like you to look at the lists below.

1. 1ST column - please tick the top 5 types of data you would like to receive feedback on and why.

2. 2nd column – please rank in order the denominators you would like to be used to determine the error rate. Why?

NUMERATOR – **please tick top 5**<br>Types of prescribing error (irrespective of actual or potential patient harm)	DENOMINATOR – **please rank (1 = most preferred info, 7 = least preferred info)**
☐ Allergy<br>☐ Omission of medication<br>☐ Unnecessary prescribing<br>☐ Clinical contraindications<br>☐ Dosing errors<br>☐ Formulation errors<br>☐ Wrong route errors<br>☐ Lack of clear directions for administration<br>☐ Technical errors (e.g. no max dose)<br>☐ Duration of treatment errors<br>☐ Miscellaneous<br>☐ Other (please state):	☐ No. of NEW medications prescribed (any time)<br>☐ No. of NEW medications prescribed 9am – 5pm<br>☐ No. of NEW medications prescribed out of hours<br>☐ No. of NEW medications prescribed (by day of week)<br>☐ No. of patients under the doctor's care (by consultant)<br>☐ No. of patients in which the doctor had prescribed at least one drug (irrespective of consultant/specialty/ward)<br>☐ Other (please state):

Did you receive feedback in the past via a different way than the methods discussed in this interview?

Is there anything else you would like to add?

Thank you for participating on this interview. Your contribution is very much appreciated

Appendix 2 Interview Topic Guide

Example 1

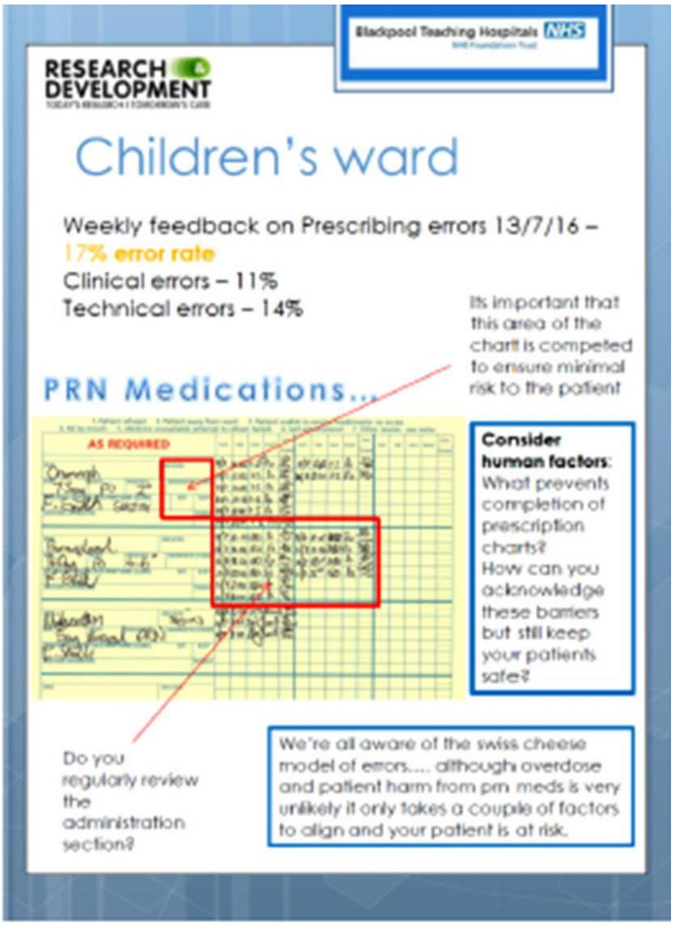

Appendix 2 Interview Topic Guide

Example 2

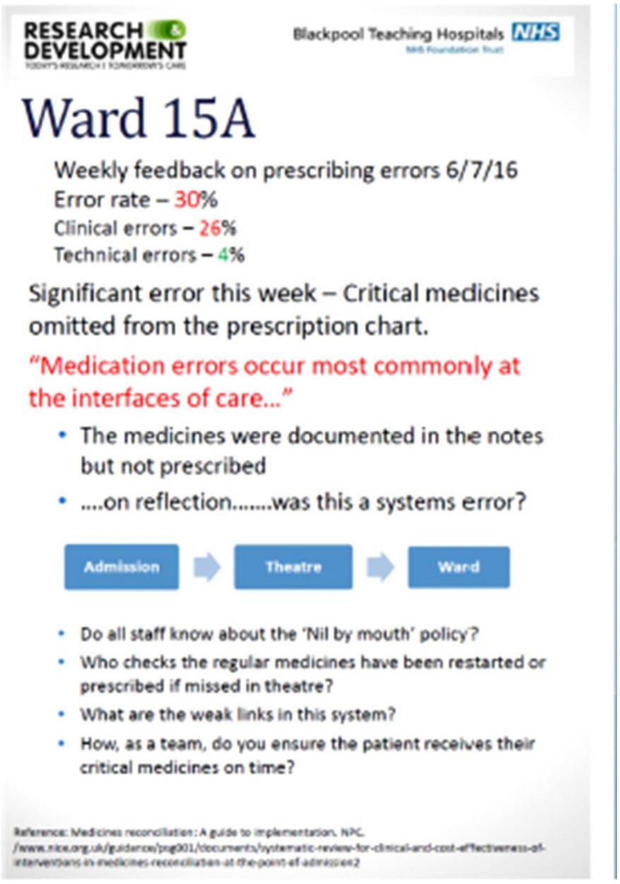

Appendix 2 Interview Topic Guide

Example 3

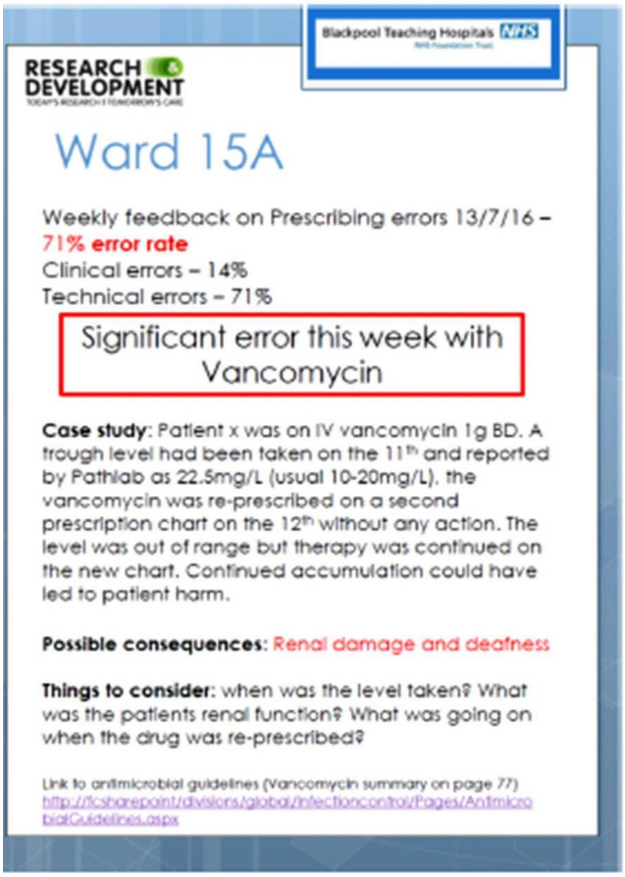

Appendix 2 Interview Topic Guide

Example 4

Western Sussex Hospitals Standard Process for Routine Ward Round
Considerative Checklist for Complete Patient Review

The purpose of this process is to ensure that the team "does it all" for all the patients during the round
Make one member of the team the "Safety Checker" who uses this checklist before leaving each patient.
The checker must highlight anything omitted, speak up and get it done!
Key = ▭ these sections must be checked in all patients, tick white boxes only when indicated

Date	Checker's Name	Checker's Status	Signed	Clinical Team	Type of Round
…/…./2015					**Routine**
Start time	Finish time	Number of Doctors	**Total number of patients**		
			**New**	**Review**	**No of wards**

**Aspect of Care**										
**Patient Initials**										
**Bed number**										
**Preparatory Discussions**	**Preparation Before Going to the Bedside**									
**Review Diagnoses**										
**Checked New Results**										
Clinical Thinking										
**Clinical Frailty?**										
**Report from Nurse?**										
**Consultation**	**Bedside Patient Consultation**									
**Nurse present?**										
**Hand hygiene?**										
**Introductions?**										
**Wristband check?**										
**Ask and Listen?**										
**Charts**	**Check All Relevant Bedside Charts**									
**Write the NEWS Score**										
**Drugs Chart checked?**										
On iv Fluid? Review chart										
Diabetic? Glucose levels										
**Planning**	**Decide on blood tests, radiology, Discuss Discharge Date, Ceiling of Care**									
Agree future tests?										
EDD discussed?										
MRFD?										
**7 Point Safety Check**	**7 Point Safety Check to Reduce Avoidable Harm**									
Pain or discomfort										
Eating and Drinking										
Bowel Function										
Urine / catheter?										
Cannula and iv lines										
Pressure area care										
Change VTE Rx?										
**All 7 points checked?**										
**Documentation**	**Consultant Inspects and Counter Signs Today's Notes**									
**Notes signed by Consultant?**										
**Summed Up to Patient?**										
**Reported Plan to Nurse?**										

© Dr G Caldwell January 2011

H:_1_Gordon_Assessment_and_Feedback_1Ward Round Pack_SOP Routine Ward Round 21150217.docx

Appendix 2 Interview Topic Guide

Example 5

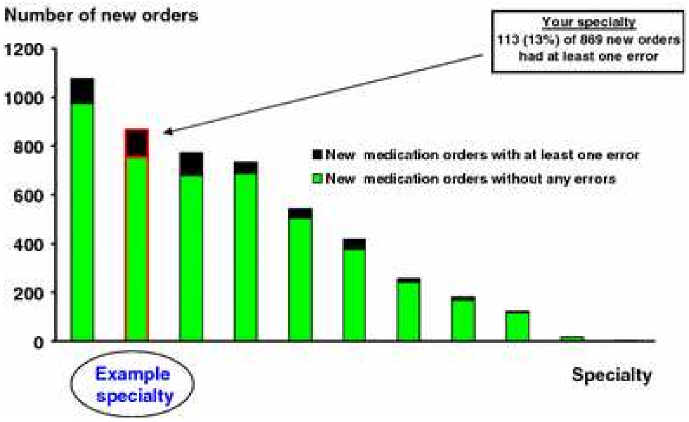

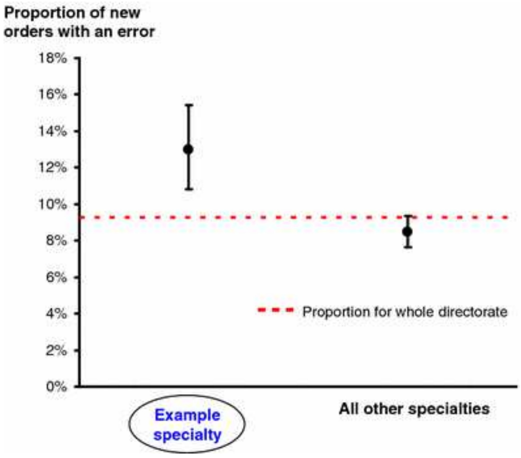

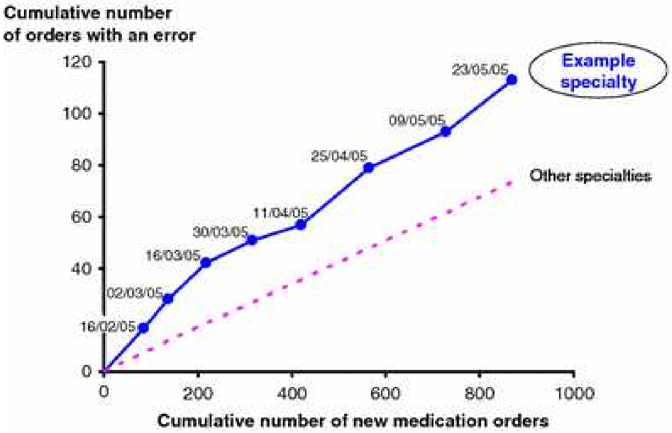

Appendix 2 Interview Topic Guide

Example 6

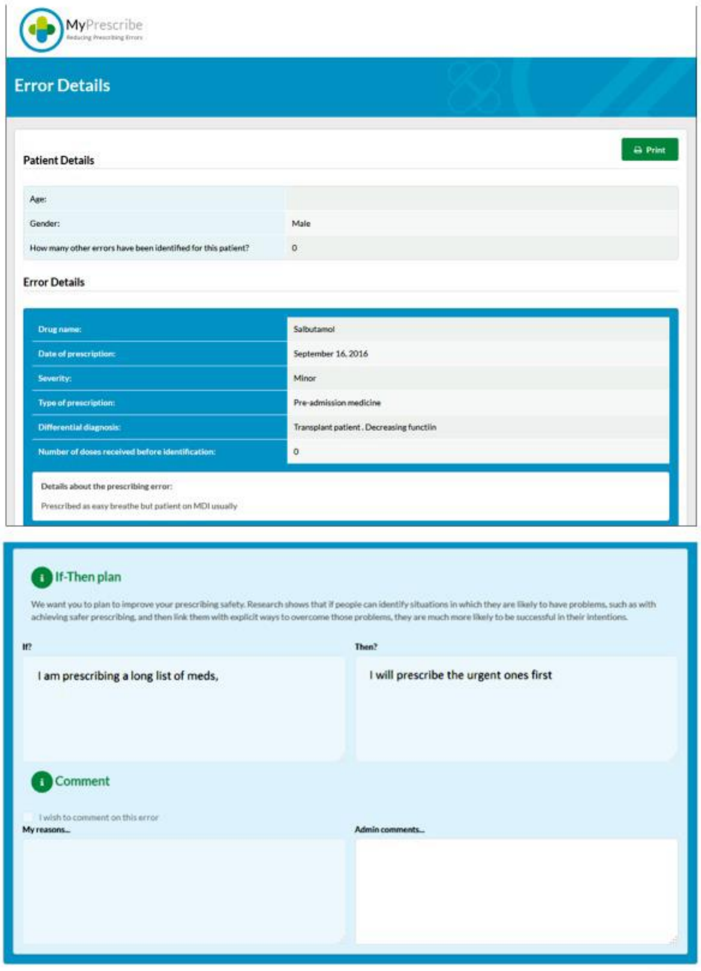

Supplement: Supplementary data [file bmjopen-2021-056221supp002.pdf]
