# Supplementary material for: Learning from electronic prescribing errors: a mixed methods study of junior doctors’ perceptions of training and individualised feedback data
Source: BMJ Open. 2022 Dec 21;12(12):e056221. doi: 10.1136/bmjopen-2021-056221 (PMC9772675; doi:10.1136/bmjopen-2021-056221)
Supplement: Supplementary data [file bmjopen-2021-056221supp003.pdf]

Appendix 3 Thematic Framework

Theme	Sub-theme	Codes
**Nature of individualised feedback data**	*Timing of feedback*	Timely feedback especially for significant errors
		Weekly feedback appropriate for minor or general errors
		Timely feedback really important
		Timely feedback to optimise recall and context – weekly or fortnightly
		Weekly feedback
		Timely personal feedback e.g. weekly
		Team every 2 weeks
		Timely feedback – weekly or fortnightly
	Characteristics of feedback	Feedback not helpful if too generic
		Tailoring the feedback e.g. selecting the most common error or significant error in that report
		Positive as well as negative feedback
		Keep feedback brief
		Short and personalised report
		Feedback needs to be specific
		Non-judgemental
		Qualitative feedback better than quantitative
		Needs to be motivational
	Content format	Combination of graphs and text
		Combination of content:
		Text poster - weekly feedback error rate + picture of example drug chart
		Text poster – weekly error rate + tips/questions for improvement
		Overview of prescribing error rate but less frequently
		Mixed feelings about error rates
		Poster with weekly error rate and example drug chart highlighting mistake
		Useful formats:
		Text poster – weekly error rate + tips/questions for improvement

Appendix 3 Thematic Framework

		Text poster – significant error highlighted with case study
		Basic qualitative info would be helpful for context
**Context of feedback**	*Relationship with feedback provider*	Feedback in person from the person correcting the mistake is more helpful
		Not received formal feedback before
		Mistakes corrected by pharmacist and feedback usually casual
		Specialised pharmacist can provide context
		From anonymised source
	Importance of error	Feedback on significant errors – actual or potential harm to patients
		Prescribing tips sometimes helpful but not always significant
		Recognised importance of major and minor errors for safe patient care
		Minor errors indicate ‘laziness’
		Feedback focussed on serious errors
		Especially for significant error
		Significant errors by others would be useful for learning
	Time to focus on feedback	Workload pressure – doctors not good at focussing on emails
		Implementing change is difficult – better to start at the beginning of a cohort
		Not always time to read emails
		Workload pressures
**Learning from feedback**	*Data validity*	Participant unclear about how data will be captured
		How to collect the data
		Equivocal about graphs – comparisons between teams and specialties may not be valid, minor and serious errors may not be separated
		Error rate on its own is not helpful
	Benchmarking with peers	Benchmarking against peers could be helpful but numbers might be too small

Appendix 3 Thematic Framework

		The person who made the error might feel bad though
	Generic case studies/examples	Learning from others' mistakes especially as Foundation doctors rotate specialties
		Learning from case study helpful
		Case studies helpful
		Learning from case study
	Teamworking culture	Personalised feedback but also in context of team prescribing culture
		Role modelling or bad habits from senior doctors
		Team hierarchy
		Feedback to team
		Team feedback and culture very important
		Personal relationships help with feedback e.g. ward or team pharmacist
		But if there is little existing relationship, better to receive a document
**EP-specific feedback**	*EP errors*	Electronic prescribing hard to adjust to from paper system
		There are some advantages to the paper charts not easily transferred to EP system e.g. suspending a drug, alternate day dosing, stopping medicines, holding a medication pending review
		EP errors e.g. meds being administered 'now' rather than pre-set times
	Other	Preparedness – national prescribing exam too easy
