# Supplementary material for: Learning from electronic prescribing errors: a mixed methods study of junior doctors’ perceptions of training and individualised feedback data
Source: BMJ Open. 2022 Dec 21;12(12):e056221. doi: 10.1136/bmjopen-2021-056221 (PMC9772675; doi:10.1136/bmjopen-2021-056221)
Supplement: Author's manuscript [file bmjopen-2021-056221.draft_revisions.pdf]

BMJ Open

BMJ Open is committed to open peer review. As part of this commitment we make the peer review history of every article we publish publicly available.

When an article is published we post the peer reviewers' comments and the authors' responses online. We also post the versions of the paper that were used during peer review. These are the versions that the peer review comments apply to.

The versions of the paper that follow are the versions that were submitted during the peer review process. They are not the versions of record or the final published versions. They should not be cited or distributed as the published version of this manuscript.

BMJ Open is an open access journal and the full, final, typeset and author-corrected version of record of the manuscript is available on our site with no access controls, subscription charges or pay-per-view fees (http://bmjopen.bmj.com).

If you have any questions on BMJ Open's open peer review process please email info.bmjopen@bmj.com

Learning from electronic prescribing errors: a mixed methods study of junior doctors' perceptions of training and personalised feedback

Journal:	*BMJ Open*
Manuscript ID	bmjopen-2021-056221
Article Type:	Original research
Date Submitted by the Author:	06-Aug-2021

Complete List of Authors:	Chu, Ann; Imperial College London, Faculty Education Office<br>Kumar, Arika; Imperial College Healthcare NHS Trust<br>Depoorter, Geraldine; Imperial College Healthcare NHS Trust<br>Franklin, Bryony; Imperial College Healthcare NHS Trust<br>McLeod, Monsey; Imperial College Healthcare NHS Trust

Keywords:	MEDICAL EDUCATION & TRAINING, GENERAL MEDICINE (see Internal Medicine), Adverse events < THERAPEUTICS, Health informatics < BIOTECHNOLOGY & BIOINFORMATICS, Quality in health care < HEALTH SERVICES ADMINISTRATION & MANAGEMENT

I, the Submitting Author has the right to grant and does grant on behalf of all authors of the Work (as defined in the below author licence), an exclusive licence and/or a non-exclusive licence for contributions from authors who are: i) UK Crown employees; ii) where BMJ has agreed a CC-BY licence shall apply, and/or iii) in accordance with the terms applicable for US Federal Government officers or employees acting as part of their official duties; on a worldwide, perpetual, irrevocable, royalty-free basis to BMJ Publishing Group Ltd ("BMJ") its licensees and where the relevant Journal is co-owned by BMJ to the co-owners of the Journal, to publish the Work in this journal and any other BMJ products and to exploit all rights, as set out in our licence.

The Submitting Author accepts and understands that any supply made under these terms is made by BMJ to the Submitting Author unless you are acting as an employee on behalf of your employer or a postgraduate student of an affiliated institution which is paying any applicable article publishing charge ("APC") for Open Access articles. Where the Submitting Author wishes to make the Work available on an Open Access basis (and intends to pay the relevant APC), the terms of reuse of such Open Access shall be governed by a Creative Commons licence – details of these licences and which Creative Commons licence will apply to this Work are set out in our licence referred to above.

Other than as permitted in any relevant BMJ Author's Self Archiving Policies, I confirm this Work has not been accepted for publication elsewhere, is not being considered for publication elsewhere and does not duplicate material already published. I confirm all authors consent to publication of this Work and authorise the granting of this licence.

For peer review only

Title Page:

Learning from electronic prescribing errors: a mixed methods study of junior doctors' perceptions of training and personalised feedback

Ann Chu, Arika Kumar, Géraldine Depoorter, Bryony Dean Franklin, Monsey McLeod

Corresponding author:
Dr Ann Chu, Academic Clinical Fellow, Reynolds Building, St. Dunstan's Road, London W6 8RP
Email: ann.chu@imperial.ac.uk

Abstract

Objectives: To explore the views of junior doctors towards (i) electronic prescribing (EP) training and feedback, (ii) readiness for receiving personalised feedback about EP errors, and (iii) preferences for receiving and learning from EP feedback.
Design: Explanatory sequential mixed methods study comprising phase 1 quantitative survey, followed by phase 2 interviews and focus group discussions.
Setting: Three acute hospitals of a large English National Health Service organisation.
Participants: 25 of 89 foundation year 1 and 2 doctors completed the phase 1 survey; seven participated in semi-structured interviews and five in a focus group in phase 2.
Results: Foundation doctors in this mixed-methods study reported that current feedback provision on EP errors was lacking or informal, and that existing EP training and resources were underutilised. They believed feedback about prescribing errors to be important and were keen to receive real-time, personalised EP feedback. Feedback also needed to be in manageable amounts, motivational and clearly signposting how to learn or improve. Participants wanted feedback and better training on the EP system to prevent repeating errors. In addition to personalised EP error data, they were positive about learning from general prescribing errors and aggregated EP data. However, there was lack of consensus about how best to learn from statistical data. Potential limitations identified by participants included concern about how the data would be collected and whether it would be truly reflective of their performance.

Conclusions: Junior doctors would value feedback on their prescribing, and are keen to learn from EP errors, develop their clinical prescribing skills and use the EP interface effectively. We identified specific user-centred gaps and preferences for EP technology to enable provision of real-time feedback to support learning and potentially reduce prescribing errors.

For peer review only

Article summary: Strengths and limitations of this study

- A key strength is the sequential explanatory mixed methods approach that allowed triangulation of survey responses with more in-depth qualitative data.
- All foundation doctors in the organisation were invited to participate; this enabled capture of context-specific views and experiences of electronic prescribing (EP) training and feedback that could be used to identify targets for improvement.
- Findings from a single organisation may not be generalisable to other hospital organisations, but may be useful for those using similar EP systems and providing similar types of training.

Funding statement:
This study was supported by the National Institute for Health Research (NIHR) Imperial Patient Safety Translational Research Centre (PSTRC-2016-004). AC is an NIHR funded Academic Clinical Fellow. The views expressed are those of the author(s) and not necessarily those of the NIHR or the Department of Health and Social Care.

Competing interests statement:
AC – no competing interests
AK – no competing interests
GD – no competing interests
BDF supervised a PhD student who was part funded by Cerner (a supplier of electronic health record systems) but whose work was unrelated to this study.
MM – no competing interests

Authors' contributions:
MM and BDF conceived the study and study design. Methods were developed by MM, BDF, GD, and AK. Data were collected by AK, GD, and MM and initial data analyses were conducted by GD, AK, AC and MM. Interpretation of the data was discussed to consensus by AC, BDF, and MM. The manuscript was prepared and revised by AC and MM. All authors reviewed and finalised the manuscript.

Data sharing statement:
Supplementary information is attached in the appendices. Raw data will be linked to a recommended repository e.g. Figshare.

For peer review only

Word count 3997

Background

Prescribing errors are reported to affect 7% of inpatient medication orders (1). Worldwide, medication errors comprise a global patient safety issue, costing an estimated £33 billion in healthcare expenditure (2). This is highlighted by the World Health Organization Global Patient Safety Challenge on 'Medication Without Harm' and potentially diverts resources away from service improvements, as well as reducing patient trust in the health care system (3).

Within the UK, newly qualified ('junior') doctors have previously been identified as having one of the highest error rates among prescribers, as well as doing most inpatient prescribing (4,5). The UK Foundation Programme is a 2-year mandatory training programme for newly qualified medical graduates. It is designed to provide junior doctors with the skills and professional capabilities of progressing to further specialist training**.** The Royal College of Physicians recognises that supporting junior doctors in safe prescribing requires education, practical resources and a culture of patient safety (6). Numerous studies have identified this group of junior doctors as a focus for educational interventions but to date, most have been in the context of paper-based prescribing(7).

However, the expansion of electronic prescribing (EP) in the English National Health Service (NHS) introduces an additional component for which staff require training. EP is generally advocated for reducing prescribing errors but studies have typically focussed on the technology and not the human factors or educational aspects(7). A recent systematic review highlighted that EP systems can also introduce other error types due to incomplete computerised displays, selection errors in drop-down menus, reliance on default settings and overdependence on clinical decision support (8).

Medication safety research based on theories of human error suggests that prescribing errors occur as a result of latent and environmental conditions aligning with, or contributing to, failures in defences (9). Learning from prescribing errors requires an understanding of the context, both at a system and individual level, and that feedback be provided for relevant stakeholders, including prescribers. However, past research has generally focused on measuring prescribing errors or root cause analysis rather than provision of feedback to support learning about prescribing (10).

A previous questionnaire study of junior doctors and hospital pharmacists found that the concept of receiving structured feedback including prescribing rates was generally well received (11). However, this work was conducted in the context of paper-based prescribing which required resource-intensive manual audits; this often resulted in considerable delay for feedback, and was generally impractical for routine implementation. We aimed to explore the views of junior doctors towards (i) the EP training and feedback they receive, (ii) readiness for receiving personalised EP feedback and (iii) preferences for how they would like to receive and learn from EP feedback.

For peer review only - http://bmjopen.bmj.com/site/about/guidelines.xhtml

Methods

Setting

We conducted a mixed-methods study at three large hospitals within the same English NHS organisation (Trust). The organisation has approximately 10,000 staff serving a population of one million people. EP has been active in the majority of inpatient areas since 2016. Existing EP training resources include online learning modules (generic and specialty-specific), classroom-based training, 'Quick Reference Guides' on the trust's intranet, some postgraduate teaching sessions, and support from the EP team. EP 'order sentences' with default doses were available for many commonly used medications, but other clinical decision support tools such as allergy and drug interaction checking were not in routine use at the time of the study.

Participants

All foundation doctors in their first two years of practice [Foundation Years 1 (FY1) and 2 (FY2)] at the trust (n=89) were invited to participate.

Study design

This was an explanatory sequential mixed methods study comprising two phases: phase 1 was a quantitative questionnaire delivered to all foundation doctors in February/March 2018; phase 2 was a qualitative interview and focus group study with volunteer participants from the same population in April/May 2018.

Ethics

This study met the Health Research Authority criteria for service evaluation; NHS ethics approval was not required. It was registered as a service evaluation within the trust (reference 266). The study was conducted in line with ethical research practice: ensuring voluntary participation, obtaining written consent for interviews and focus groups, and anonymising data in line with data protection regulations. There was no patient and public involvement in this research.

Recruitment

Phase 1 questionnaires were distributed online and on paper. Email circulation via postgraduate education teams ensured that all foundation doctors working in the organisation had an opportunity to participate. The email summarised the purpose of the study and included a weblink to an online version of the questionnaire. In addition, paper questionnaires were distributed at teaching sessions.

In phase 2, foundation doctors were invited to participate in interviews or focus groups, whichever they preferred and/or was able to attend, using both email and face-to-face recruitment. Ward pharmacists were asked to help promote the study and the project team also

attended teaching sessions to recruit foundation doctors. All participation was voluntary and written consent obtained for interviews and focus group.

Data collection

Phase 1 – questionnaire study

The questionnaire (appendix) comprised 65 questions. This was based on a questionnaire used in a previous study (11) but with two major adaptations: (i) questions were adapted to EP, and (ii) clinical scenarios were added. Most responses required were on five-point Likert scales ranging from 'strongly disagree' to 'strongly agree'. Where relevant, there were additional options for 'not used' or 'not available'.

Four clinical scenarios were devised as examples of prescribing errors with different levels of harm. These were based on categories from a national incident reporting system comprising 'no harm', 'low harm', 'moderate harm' to 'severe harm' (12) . The fifth category of 'death' was not included.

Phase 2 – qualitative interview and focus group study

Interviews and focus group were conducted using a pre-piloted topic guide (appendix) that was informed by the findings from phase 1. Six example prescribing error feedback formats were obtained from the published literature and shown to participants. A table of EP error types (e.g. allergies, dosing errors) was also used as a prompt for discussion. Handwritten notes were recorded for both the focus group and the interviews, which included some verbatim comments from participants.

Data analysis

Phase 1 quantitative data were summarised using descriptive statistics, using Microsoft Excel. Positive responses ('agree and strongly agree') and negative responses ('disagree and strongly disagree') were combined when reporting the data in the results section.

Phase 2 interview and focus group notes were transcribed and then analysed thematically using an inductive approach. Using a triangulation approach, analysis of quantitative and qualitative data was integrated by two researchers and discussed to consensus by wider research team.

Results

In the phase 1 quantitative study, 25 of 89 (28%) foundation doctors submitted responses. Respondents comprised 14 females (56%), 8 males (32%) and 3 whom did not disclose their gender. The majority were FY1 (n=16), six were FY2 (n=6) and three did not indicate their grade. Respondents were from 14 different medical schools.

In the phase 2 qualitative study, 12 participants took part in either a focus group (n=7) or an individual interview (n=5). Eight were FY1 and four were FY2.

Phase 1: Quantitative survey data

Current level of EP training and feedback

Foundation doctors had varied experiences of EP and associated training resources [figure 1]. They were equivocal as to whether EP reduced the number of prescribing errors that led to patient harm (12/25 agreed). Similarly, about half were satisfied with the training that had been provided at induction (13/25). More than half were satisfied with the availability of resources to enable them to prescribe safely (18/25). However, there was lack of awareness and/or use of specifically tailored training resources. Some reported not having received EP classroom training (7/25) and 13/25 reported not using the specialty-specific training modules. The majority reported not receiving the training provided as part of the postgraduate education sessions (14/25) or not having used the 'Quick Reference Guides' and crib sheets available on the intranet (17/25). However, in free text responses, respondents perceived that training on the EP interface would be useful, and that better understanding of the EP system might help prevent errors.

The majority of respondents said they were not always informed of their own prescribing errors (17/25) and that this was of concern. If they did receive feedback, this was generally in an unplanned or unscheduled way (21/25). Free text responses reconfirmed that "little" or "rare" feedback was received. Junior doctors stated they would seek EP help from their peers (23/25), pharmacists (22/25) or more senior doctors (12/25). However, they said they generally did not ask nurses (6/25) or consultants (2/25).

Readiness for receiving EP feedback

Most respondents (23/25) believed receiving feedback about prescribing errors was important and that being aware of their own errors would help improve their prescribing [figure 2]. They believed receiving formal feedback on prescribing errors would reduce future errors (22/25). Foundation doctors wanted to know about all prescribing errors they made (22/25), including minor errors (19/25) and confirmed this when presented with example clinical scenarios covering 'no harm' (18/25) and 'low harm' (22/25).

For peer review only

Preferences for receiving and learning from personalised EP feedback

Participants were keen for personalised feedback to be timely; they supported a variety of methods including verbal (19/25), instant messaging (22/25) and/or EP system emails (18/25) [Figure 3].

Respondents were receptive to the idea of receiving a regular report (20/25) and the ability to track errors made over time (19/25). They were keen to review personal EP errors with a pharmacist (19/25). However, there was lack of consensus about doing this with their peers (7/25 agreed, 9/25 disagreed) and opposition to reviewing EP errors with a consultant (6/25 agreed; 13/25 disagreed).

Participants were also keen to learn from others' prescribing errors that were relevant to their practice (20/25). This information could be received in several ways, including on the ward (14/25), in existing training sessions (17/25) or new dedicated sessions (13/25). They were also receptive to receiving a regular summary of common prescribing errors within their specialty (20/25), rare but serious errors in their specialty (18/25), and in the organisation (15/25).

Phase 2: Qualitative interview and focus group data

Preferences for receiving and learning from EP feedback

Participants discussed both general prescribing feedback and their perceptions of EP-specific feedback. Participants' preferences for receiving and learning from prescribing feedback were grouped into four themes: **(i) nature of personalised feedback, (ii) context of feedback, (iii) learning from feedback, and (iv) EP-specific feedback.**

(i) Nature of personalised feedback

The most consistent theme among participants was the need for timely feedback when specific errors were detected. This was especially important for significant clinical errors. Due to working patterns, the original prescriber might not be aware of their prescribing error at all. Feedback on a specific error within a week or two was required to optimise recall of the patient's clinical situation and the doctor's working context. More timely feedback using the EP system was seen as an advantage compared to feedback based on paper drug charts and manual audit.

Participants wanted personalised feedback to be specific, tailored and concise. They also focussed on the learning aspect rather than the error itself and wanted feedback to be non-judgemental, balanced and motivational, especially if delivered by email.

"It would be nice to have a headline that says something like 'congratulations, you only made two errors this past month' – give some positive feedback as well as information about prescribing error" (FY2, Interview 2).

"Error rate sounds a bit judgey [sic] and feels like an attack, feels like you've been watched" (FY1, Focus group)

In terms of format and content, there was no consensus on preferences around the example reports shared. Instead, a combination of text, illustrations, graphs and case studies were generally considered to be useful.

Context of feedback

Although participants were generally positive about receiving feedback on EP errors, this depended on the context in which it was given and received. An important factor was the feedback provider. EP feedback in a report from an anonymised source was perceived to be potentially problematic due to workload pressures and limited time to focus on emails. Most participants had received verbal feedback from their ward pharmacist who had corrected an error and were comfortable with this if they had worked with them before. There was a degree of trust and confidence in particular with specialist clinical pharmacists (e.g. renal, paediatrics) as they were perceived to understand the nuances of prescribing for their patient cohort and were able to better highlight clinically relevant errors. However, participants raised concern about the potential workload for their ward pharmacist if more regular formal feedback was to be delivered due to the time required for data collection and also being able to reciprocate with time to receive feedback.

"Sometimes pharmacists will say "oh you do know that you got this wrong" but it's done casually" (FY2, interview 1).

There was consensus from participants that feedback on more significant prescribing errors (i.e. those associated with potential or actual 'major harm') was very important. The value of feedback for less serious errors was less convincing as participants perceived that they would not have time to focus on all errors. When given a list of example error types, participants ranked prescribing a drug to which a patient has an allergy as the most important error to receive feedback on. Clinical contraindications, omissions and unnecessary prescribing were also prioritised for feedback. However, dosing errors and formulation errors were not.

(ii) Learning from feedback

Participants acknowledged that learning from prescribing feedback could take place at an individual level, in a group setting or even at organisational level.

There were mixed feelings about learning from data such as baseline error rates; some participants perceived this to be helpful for context, whilst others felt that statistics (especially in isolation) would not be as helpful for learning in comparison to narrative feedback. Similarly, benchmarking statistics with peers had a mixed response; some participants valued the quantitative aspect while others were unclear about how this would advance their learning and were afraid of being judged.

For peer review only

"Error rates is like a percent on your exams…It doesn't say anything, and you can't learn from it" (FY1, focus group)

However, there was general agreement that learning about prescribing errors in the form of case studies was a helpful educational tool. Learning about errors made by peers in a group setting was useful, especially as foundation doctors rotated specialties. However, it was noted that reporting on real EP errors publicly needed to be done carefully and anonymously, as there was concern about having a negative impact: *"…it might break the team spirit" (FY1, focus group).*

One participant was clear that all prescribing errors were significant for patient safety and that accepting minor errors was suggestive of professional 'laziness'. Instead, they felt that the organisation should be learning from all types of errors and cultivating good prescribing practice:

"We as a hospital are lazy… for example, in giving the indication for medications" (FY2, interview 4).

The team prescribing culture was felt to be important, as junior doctors were influenced by senior decision makers, particularly in terms of bad habits or prescribing outside the protocol. Moreover, prescribing decisions were often made by the consultant but enacted by the junior doctors. Therefore, one participant suggested providing feedback to the team in general as well as individuals.

"The junior doctors are a lot influenced by the senior doctors and there is a hierarchy, sometimes I want to say something… but it's in some way difficult to correct them. But anyway, I just say it… because patient safety is the priority… It's the job of the entire team to look at the drug chart and review it" (FY1, interview 4).

In addition, error rates on its own might not be helpful for comparing between specialties with different prescribing cultures, therefore reducing the validity of the data.

"Graphs are a nice idea in some ways… but if you are in a specialty and you are not making many errors, it's weird to be like oh well, I do way better than the other specialties… how serious are these errors on the graphs? If you do badly it's embarrassing for the team, but it can be a good thing for some… maybe they are going to do more to do their best" (FY2, interview 5)

(iii) EP-specific feedback

Participants perceived that the EP system architecture was capable of providing greater prescribing support than paper prescribing and there was a potential for EP data to be used for feedback. Participants wanted to have a better understanding of the EP system to avoid repeating errors and queried how EP error data would be accurately collected and judged. There was anxiety about being monitored and that automated EP data might not accurately reflect their abilities.

For peer review only

There were some examples of participants who had difficulties transitioning from paper-based system and had identified EP-specific challenges such as prescribing complex scheduling times, re-scheduling drug administration times, and suspending medication doses. Specifically, participants found that the default settings for standard drug administration times made it more difficult to prescribe accurately, exposing them to risks of making EP errors. Participants were keen to know how to use the EP system better in these situations and how the system could be adapted to be more intuitive.

Some participants also suggested that the national student prescribing safety assessment was not a good marker for preparedness and was not helpful for EP skills: *"it's very different from actually using the system" (FY2, interview 5).*

For peer review only

Discussion

Key findings

Foundation doctors in this mixed-methods study reported that current feedback provision on EP errors was lacking or informal, and that existing EP training and resources were underutilised. They believed feedback about prescribing errors to be important and were keen to receive real-time, personalised EP feedback. Feedback also needed to be in manageable amounts, motivational and clearly signposting how to learn or improve. Participants wanted feedback and better training on the EP system to prevent repeating errors. In addition to personalised EP error data, they were positive about learning from general prescribing errors and aggregated EP data. However, there was lack of consensus about how best to learn from statistical data. Potential limitations identified by participants included concern about how the data would be collected and whether it would be truly reflective of their performance.

Interpretation

This study supports the findings of previous research that foundation doctors do want feedback on prescribing errors (11). With the evolution of EP technology, there is hope that this could be delivered in a timely, personalised and digitised format to optimise learning. However, provision of EP error information on its own would not be adequate to promote reflective practice as foundation doctors would be more concerned about being performance managed, as highlighted by others (13). Contextualising EP errors using illustrative case studies and having the opportunity to debrief with a pharmacist were welcomed. Enabling novice prescribers to have enough time and cognitive space to both consider the feedback and formulate an action plan were identified as potential challenges. One example of an electronic feedback tool involving personalised learning plans has been described by others (14).

Interview participants discussed 'feedback', but it was not always clear whether they were distinguishing between general prescribing feedback and provision of EP data. This may partly be due to the scoping nature of the study - participants did not have direct experience of this type of EP feedback mechanism and so were considering a hypothetical situation. In addition,

FY1 doctors may not have direct experience of prescribing in any other setting. It is therefore possible that participants and researchers were discussing different aspects of 'feedback' but using the term interchangeably to mean different things. This has also been found in the educational literature, where there is well-recognised incongruence between learners and teachers as to the definition, quantity and quality of feedback given and received (15).

Strengths and limitations

The main strength of this study came from combining quantitative and qualitative data collection methods. This gave us the opportunity to explore the research question from different perspectives. The survey was completed by more participants and captured a broader range of respondents, while the interviews and focus group gave us opportunity to explore the issues in more depth. Eliciting the challenges and anxieties perceived by foundation doctors was a strength of the qualitative data, as there can often be an implementation gap if these issues are not recognised prior to development of new tools or systems. The multidisciplinary nature of the research team (doctor and pharmacists, with medical education and medication safety expertise) was also a strength as we were able to contribute different perspectives to the data analysis from the context of our professional experience.

Limitations of this study include the low response rate and small sample size from a single organisation, which limits generalisability. There may be participation bias, particularly with interviews and focus groups, as respondents may be more likely to be interested in the study concept. They may also have provided socially desirable responses rather than their true viewpoints. We explored participants' perceptions rather than conducting a direct evaluation in practice. Participants therefore made hypothetical assumptions about the capability of the EP system and provision of EP data. The examples that participants were shown in the interviews were based on the literature and thus were mainly paper-based.

Implications for practice

This data informs future work for developing personalised, digital EP feedback data for prescribers. Feedback needs to be timely, concise and relevant, with potentially a blended approach in terms of providing data and example case studies both verbally and electronically. Situational factors should be acknowledged, and feedback should be supportive and non-judgemental. Consideration should be given to the role of pharmacists in providing feedback, and allocating sufficient time for prescribers to focus on the feedback delivered, prioritising the relative importance of EP errors and support formulation of action plans. Other learning points include improving awareness and engagement of EP training opportunities, changing the focus to prescribing accuracy (rather than on errors), maximising opportunity for reflective practice (not just providing information) and enabling individuals to contribute to organisational learning and improvements (for example, reporting EP system defaults). A challenge to achieving this in practice is identifying how this work might fit within existing pharmacy roles and what further digital resources might be required to maximise sustainability.

For peer review only

Future research

Future research should focus on the evaluation of EP feedback mechanisms and how to integrate this into clinical practice for novice prescribers. The feasibility, acceptability and utility of these tools should be considered and appropriate mechanisms for providing context and promoting learning put into place. There may also be unintended consequences and/or challenges of introducing a novel EP feedback interface which should therefore be captured. Our study focused on junior doctors as the main prescriber in hospitals, but in the UK, independent nurse and pharmacist prescribers also have an important role and provision of prescribing feedback to all types of prescribers should be explored.

Conclusion

The digital transformation of hospital inpatient prescribing presents a timely opportunity to develop, implement and evaluate a personalised prescribing feedback and learning initiative to reduce prescribing errors. Foundation doctors in this study would value feedback on their prescribing, are keen to learn from errors, develop their clinical prescribing skills and use the electronic interface effectively. Our findings suggest a range of tailored, user-centred options would be a positive strategy to enhance the value of providing personalised EP feedback for novice prescribers. We found a demand for EP technology to enable provision of real-time feedback to support learning, thus potentially reducing prescribing errors. The next steps are to develop a feasible approach for extracting such data, providing EP feedback and evaluating its utility and effectiveness in practice.

For peer review only

References

1. Lewis PJ, Dornan T, Taylor D, Tully MP, Wass V, Ashcroft DM. Prevalence, incidence and nature of prescribing errors in hospital inpatients: A systematic review. Drug Saf. 2009;32(5):379–89.

2. Donaldson LJ, Kelley ET, Dhingra-Kumar N, Kieny MP, Sheikh A. Medication Without Harm: WHO's Third Global Patient Safety Challenge. Lancet [Internet]. 2017;389(10080):1680–1. Available from: http://dx.doi.org/10.1016/S0140-6736(17)31047-4

3. Society MP. The rising cost of clinical negligence: who pays the price? 2017.

4. Dornan T, Ashcroft D, Heathfield H, Lewis P, Miles J, Taylor D, et al. An in-depth investigation into causes of prescribing errors by foundation trainees in relation to their medical education: EQUIP study. London; 2009.

5. Ryan C, Ross S, Davey P, Duncan EM, Francis JJ, Fielding S, et al. Prevalence and causes of prescribing errors: The PRescribing Outcomes for Trainee Doctors Engaged in Clinical Training (PROTECT) study. PLoS One. 2014;9(1):1–9.

6. Ferner R. Supporting junior doctors in safe prescribing Supporting junior doctors in safe prescribing. R Coll Physicians. 2017;

7. The Health Foundation. Evidence scan: Reducing prescribing errors. 2012;(April):36.

8. Brown CL, Mulcaster HL, Triffitt KL, Sittig DF, Ash JS, Reygate K, et al. A systematic review of the types and causes of prescribing errors generated from using computerized provider order entry systems in primary and secondary care. J Am Med Informatics Assoc. 2017;24(2):432–40.

9. Puaar SJ, Franklin BD. Impact of an inpatient electronic prescribing system on prescribing error causation: A qualitative evaluation in an English hospital. BMJ Qual Saf. 2018;27(7):529–38.

10. Hollnagel E. From Safety-I to Safety-II : A White Paper.

11. Bertels J, Almoudaris AM, Cortoos PJ, Jacklin A, Franklin BD. Feedback on prescribing errors to junior doctors: Exploring views, problems and preferred methods. Int J Clin Pharm. 2013;35(3):332–8.

12. NHS. NRLS Organisation patient safety incident workbook. 2017;(September).

13. Redwood S, Ngwenya NB, Hodson J, Ferner RE, Coleman JJ. Effects of a computerized feedback intervention on safety performance by junior doctors: Results from a randomized mixed method study. BMC Med Inform Decis Mak. 2013;13(1):1–10.

14. Keyworth C, Hart J, Thoong H, Ferguson J, Tully M. A Technological Innovation to Reduce Prescribing Errors Based on Implementation Intentions: The Acceptability and Feasibility of MyPrescribe. JMIR Hum Factors. 2017;4(3):e17.

15. Ajjawi R, Regehr G. When I say … feedback. Med Educ. 2019;53(7):652–4.

For peer review only

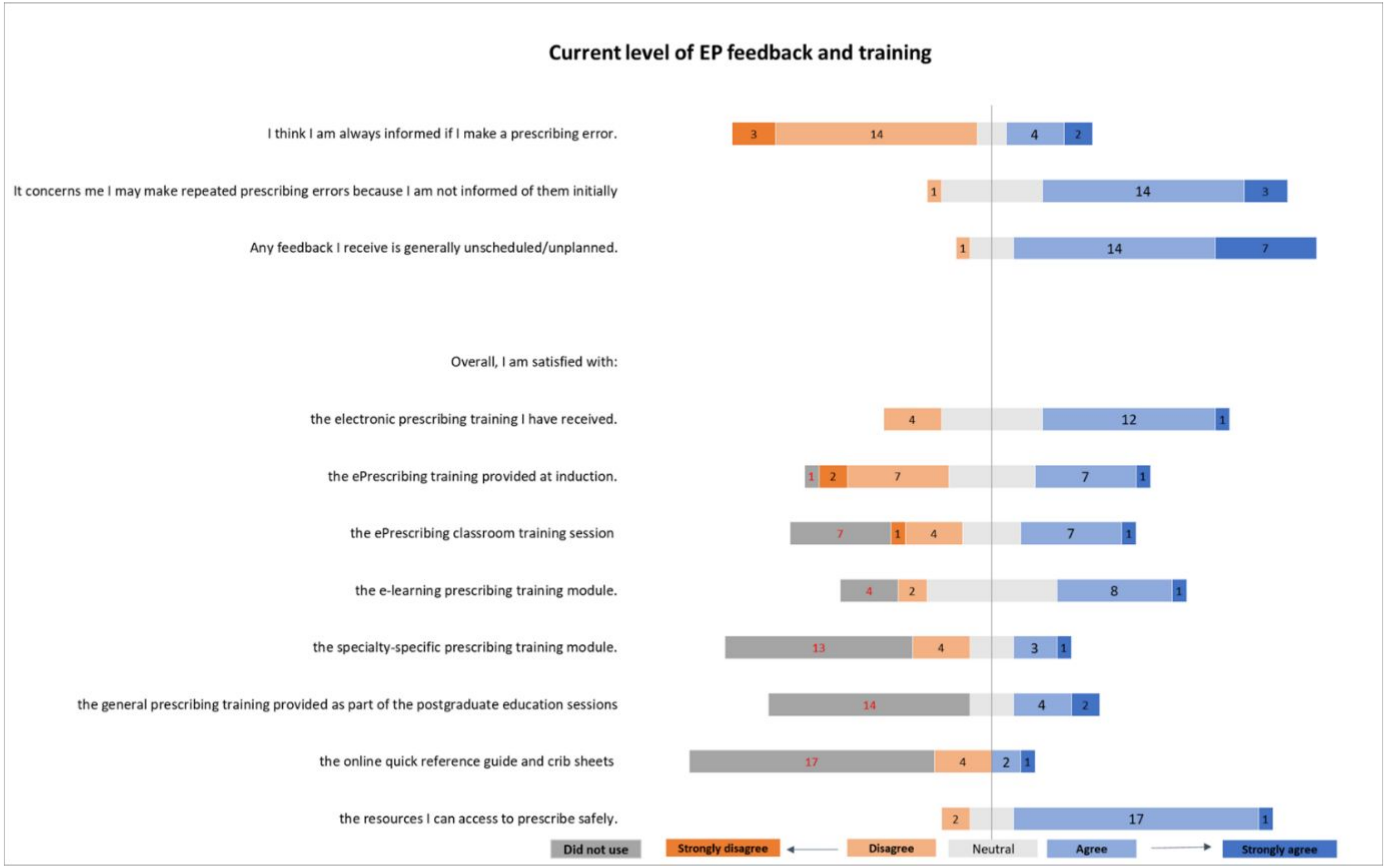

Figure 1. Current level of electronic prescribing (EP) feedback and training (numbers correspond with number of respondents, total 25 participants)

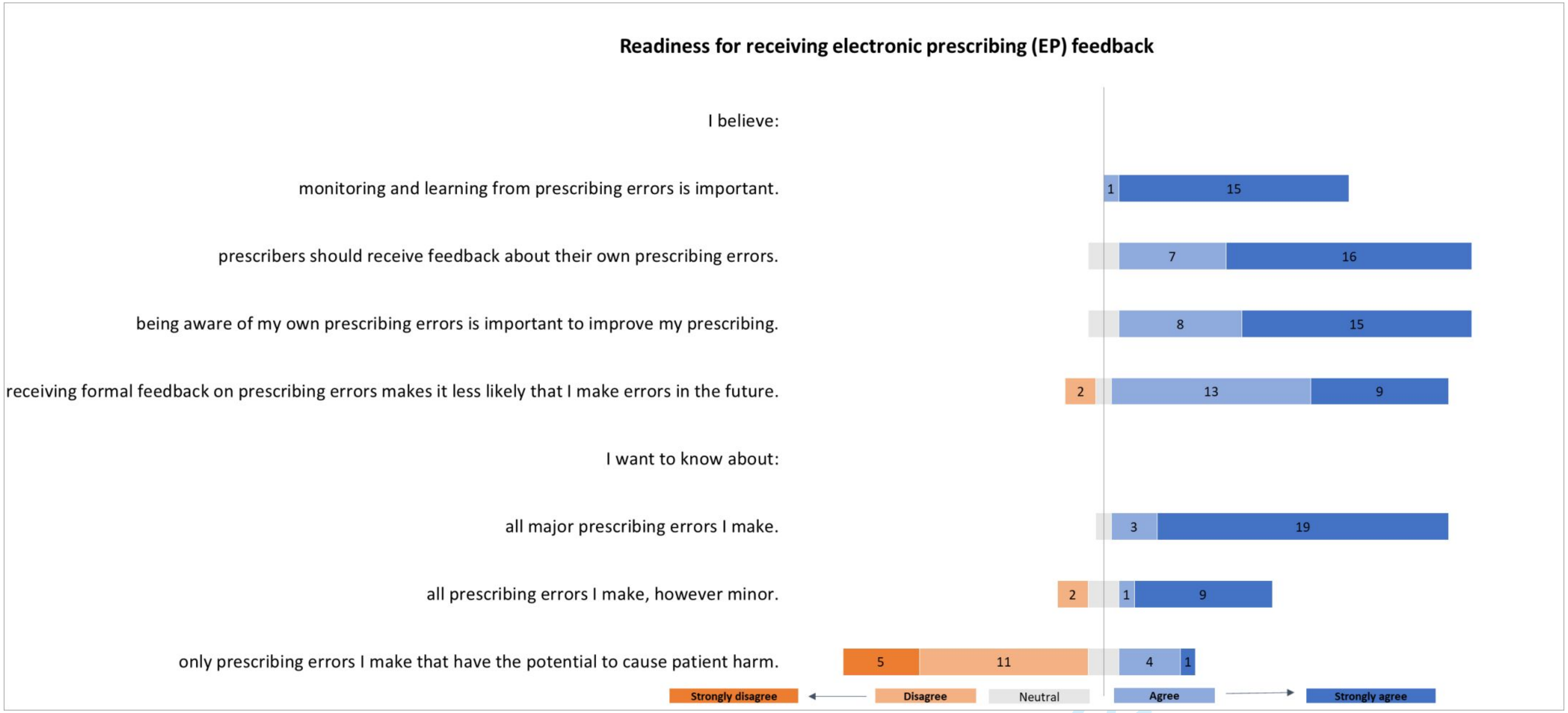

Figure 2. Readiness for receiving electronic prescribing (EP) feedback (numbers correspond with number of respondents, total 25 participants)

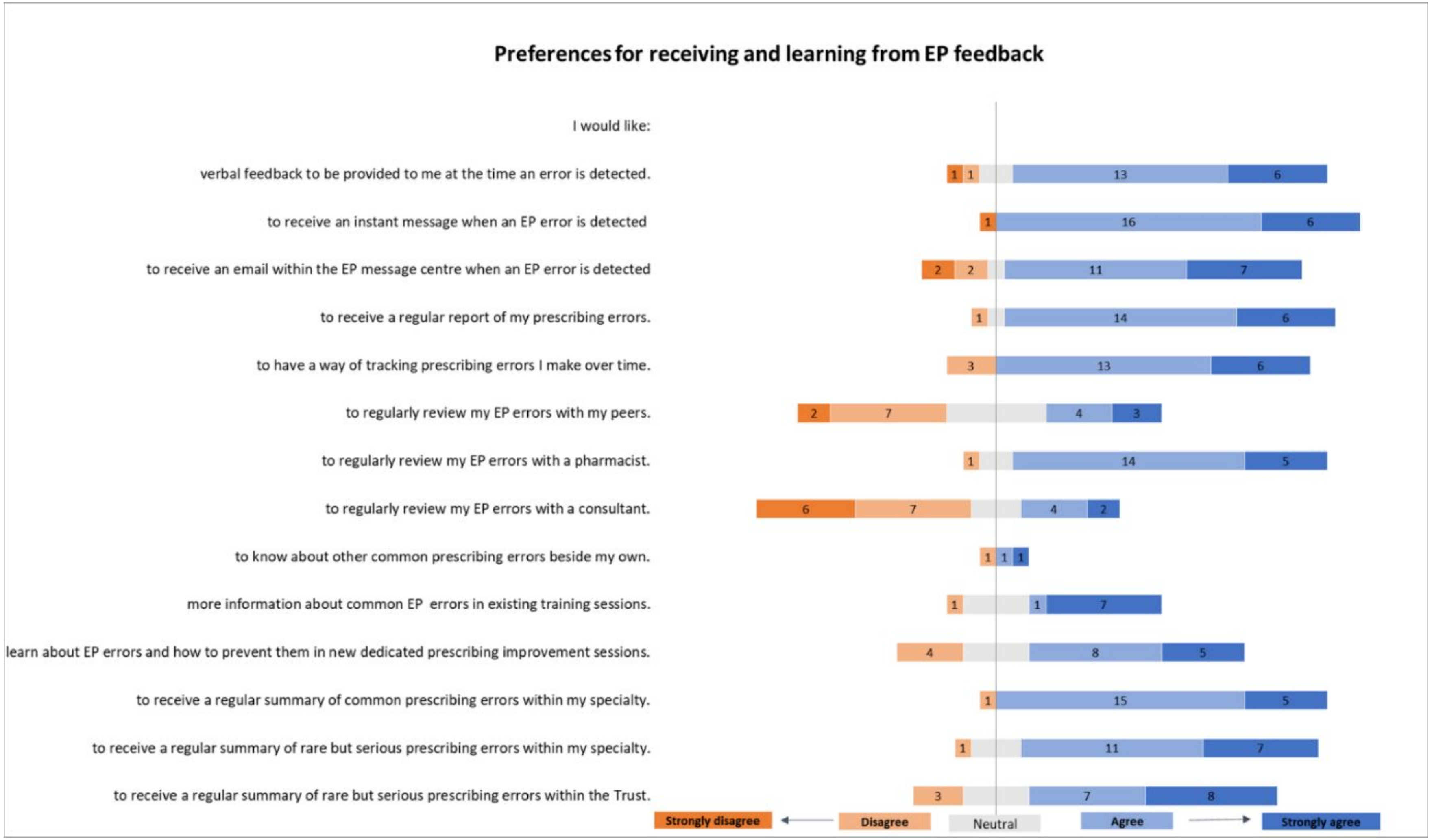

Figure 3. Preferences for receiving and learning from electronic prescribing (EP) feedback (numbers correspond with number of respondents, total 25 participants)

Appendix 1:

Junior doctors' prescribing safety survey

Improving safety and effectiveness of electronic prescribing – how can training and learning from errors be improved?

Invitation: **All foundation year 1 and 2 doctors** at Imperial College Healthcare NHS Trust.

This questionnaire forms part of a study that aims to improve prescribing safety in hospitals with electronic prescribing systems. It centres on how the Trust can help make it easier to prescribe safely and effectively, particularly in the way in which electronic prescribing training and feedback about errors are currently provided. It also asks about your preferred methods for receiving feedback on your prescribing errors and learning from prescribing errors. Participation is voluntary, and all information will be treated confidentially. **If you have any questions, please contact monsey.mcleod@nhs.net on behalf of the Imperial Prescribing Feedback team.**

In the following questions, please select the answer that best represents your opinion by circling it.
(**SD**= Strongly Disagree, **D**= Disagree, **N**= Neutral, **A**= Agree, **SA**= Strongly Agree)

A. YOUR PRESCRIBING AND FEEDBACK EXPERIENCE

I am confident in prescribing the correct and appropriate **non-intravenous** medications for patients via Cerner.		SD	D	N	A	SA
I am confident in prescribing the correct and appropriate **intravenous** medications for patients via Cerner.		SD	D	N	A	SA
I would prefer to use a paper drug chart.	Never used	SD	D	N	A	SA
I think I am always informed if I make a prescribing error.		SD	D	N	A	SA
It concerns me I may make repeated prescribing errors because I am not informed of them initially.		SD	D	N	A	SA
I think receiving formal feedback on prescribing errors makes it less likely that I make errors in the future.		SD	D	N	A	SA
Any feedback I receive is generally unscheduled/unplanned.		SD	D	N	A	SA

B. QUALITY IMPROVEMENT

I believe electronic prescribing has reduced the number of prescribing errors that result in actual patient harm.	Don't know	SD	D	N	A	SA
I believe monitoring and learning from prescribing errors is important.		SD	D	N	A	SA
I believe prescribers should receive feedback about their own prescribing errors.		SD	D	N	A	SA
I believe being aware of my own prescribing errors is important to improve my prescribing.		SD	D	N	A	SA

C. TRAINING

Overall, I am satisfied with electronic prescribing training I receive.		SD	D	N	A	SA
I am satisfied with the following: ePrescribing training provided at induction.	Not received	SD	D	N	A	SA
I am satisfied with the ePrescribing classroom training session provided by the Trust Information Communications and Technology (ICT) team.	Not received	SD	D	N	A	SA
I am satisfied with the e-learning prescribing training module.	Not received	SD	D	N	A	SA
I am satisfied with the specialty-specific prescribing training I receive.	Not received	SD	D	N	A	SA

For peer review only - http://bmjopen.bmj.com/site/about/guidelines.xhtml

I am satisfied with the prescribing training provided as part of the postgraduate education sessions for F2/CMT (covers general prescribing safety including electronic prescribing tips).	Not received	SD	D	N	A	SA

D. RESOURCES

Overall, I am satisfied with the resources I can access to prescribe safely.		SD	D	N	A	SA
When relevant, I like to use the 'EPA quick reference guide' and crib sheets on the Source to help me prescribe.	Not used	SD	D	N	A	SA
In general, I mostly contact the pharmacy EPA team for technical prescribing queries relating to the Cerner system.	Not used	SD	D	N	A	SA
In general, I mostly ask **nurses** for help whilst prescribing (for technical Cerner-specific queries or medication-related queries).	Not used	SD	D	N	A	SA
In general, I mostly ask the **ward pharmacist** for help whilst prescribing (for technical Cerner-specific queries or medication-related queries).	Not used	SD	D	N	A	SA
In general, I mostly ask **my peers** for help whilst prescribing (for technical Cerner-specific queries or medication-related queries).	Not used	SD	D	N	A	SA
In general, I mostly ask a **CMT** for help whilst prescribing (for technical Cerner-specific queries or medication-related queries).	Not used	SD	D	N	A	SA
In general, I mostly ask the **consultant** for help whilst prescribing (for technical Cerner-specific queries or medication-related queries).	Not used	SD	D	N	A	SA
In general, I mostly ask nurses for help whilst prescribing **out-of-hours**	Not used	SD	D	N	A	SA
In general, I mostly ask the on-call pharmacist for help whilst prescribing **out-of-hours**	Not used	SD	D	N	A	SA
In general, I mostly ask my peers for help whilst prescribing **out-of-hours**	Not used	SD	D	N	A	SA
In general, I mostly ask a CMT for help whilst prescribing **out-of-hours**	Not used	SD	D	N	A	SA

E. PROSPECTIVE FEEDBACK (specific error) INCLUDING EXAMPLE SCENARIOS

I want to know about all **major** prescribing errors I make.	SD	D	N	A	SA
I want to know about **all** prescribing errors I make, however minor.	SD	D	N	A	SA
I **ONLY** want to know about prescribing errors I make that have the potential to cause patient harm.	SD	D	N	A	SA
I would like verbal feedback to be provided to me at the time the error is detected.	SD	D	N	A	SA
I would like to be able to receive an instant message when a prescribing error I've made has been detected.	SD	D	N	A	SA
I would like to be able to receive an email within the Cerner message centre when a prescribing error I've made has been detected.	SD	D	N	A	SA

I would like to receive feedback if I made this type of prescribing error:

An 82-year-old male patient with hospital acquired pneumonia was prescribed temocillin 2g IV BD and amoxicillin IV 1g TDS. He had a penicillin allergy with known anaphylaxis but his allergy status had not been completed on the electronic prescribing system. The error was detected and corrected before it reached the patient.	SD	D	N	A	SA
A 67-year-old female admitted for elective right hip replacement was started on prophylactic antibiotics at induction. Cefuroxime 1.5g IV TDS was prescribed post-op, intended for 24 hours but no stop date was prescribed. The patient received cefuroxime 1.5g IV TDS for two extra days before it was stopped. The patient has not reported any side effects from the prolonged course.	SD	D	N	A	SA
A 55-year-old male with type 2 diabetes and hypertension was admitted. One of their medications, ramipril 10mg OD, was not prescribed and the patient missed their dose for 3 days over the weekend. Their blood pressure had increased gradually and is now 190/110 mmHg.	SD	D	N	A	SA

A 11-month old girl was prescribed amoxicillin 125mg/5ml suspension twice. One was for 2.5ml TDS for one week, and the other for 5ml TDS for one week. The error was corrected before it reached the patient.	SD	D	N	A	SA

F. RETROSPECTIVE FEEDBACK (error trends specific to my own prescribing)

I would like to receive a regular summary of my prescribing errors.	SD	D	N	A	SA
I would like to have a way of tracking prescribing errors I make over time.	SD	D	N	A	SA
I would like the opportunity to regularly review my prescribing errors with **my peers**.	SD	D	N	A	SA
I would like the opportunity to regularly review my prescribing errors with a **pharmacist**.	SD	D	N	A	SA
I would like the opportunity to regularly review my prescribing errors with a **consultant**.	SD	D	N	A	SA
Feedback (review) sessions about prescribing errors of relevance to my specialty would be most valuable on the **ward**, (e.g. at handover, as part of ward round).	SD	D	N	A	SA

G. AGGREGATED RETROSPECTIVE FEEDBACK (of prescribing errors within Imperial)

I would like to know about other common prescribing errors besides my own.	SD	D	N	A	SA
I would like more information about common prescribing errors in existing training sessions.	SD	D	N	A	SA
I would like to learn about prescribing errors and how to prevent them in new dedicated prescribing improvement sessions.	SD	D	N	A	SA
I would like to receive a regular summary of **common** prescribing errors within my specialty.	SD	D	N	A	SA
I would like to receive a regular summary of **rare but serious** prescribing errors within my **specialty**.	SD	D	N	A	SA
I would like to receive a regular summary of **rare but serious** prescribing errors within the **trust**.	SD	D	N	A	SA

Please answer the following questions.
If you had enough time, what three things would make it easier for you to prescribe safely and efficiently?

What are the main concerns regarding the current feedback you receive, if any, on prescribing errors?

Sex: F / M	Grade:	Medical school qualified from:

Please email monsey.mcleod@nhs.net on behalf of the Imperial Prescribing Feedback team for any of the following:

- ☐ I would like to receive a copy of the survey results
- ☐ I am happy to be contacted to clarify my answers above
- ☐ I am happy to be contacted about future improvement projects

Thank you for completing this questionnaire.
Please return to this to the Postgraduate Medical Education team at your site (directly or internal mail).

For peer review only - http://bmjopen.bmj.com/site/about/guidelines.xhtml

Prescribing Safety Feedback Interview

Thank you for taking the time to participate this study. My name is XX and I am currently doing some research into feedback on prescribing errors within this trust.

Have you had a chance to look at the participant information sheet? Do you still have any questions? Are you happy for us to take notes? Are you happy to sign this consent form? No identifiable information is being recorded.

This interview is part of a larger study to explore the views of junior doctors and other stakeholders about providing feedback concerning prescribing errors. The interview focuses on how best to provide feedback to foundation year doctors to help make it easier to prescribe safely on the electronic system. This includes receiving feedback on prescribing errors as well as tips for how to prevent them. Participation is voluntary, and these interviews are completely anonymous.

Selection of prescribing error related feedback strategies

We know from a recent survey at this trust that FY1 and FY2 doctors do want to receive feedback about their own prescribing errors and also learn from errors elsewhere. In response to this, we are now looking to get your views on a range of different methods for receiving prescribing feedback.

PART 1

I will show you a few ways of providing feedback to support learning from prescribing errors and would like your feedback on each strategy. [ACTION: give out documents – see below].

Questions to ask while they are looking at the documents:

1. Which of these feedback reports do you prefer? Please rank in order of preference.

- *[Wait until they have put them in order of preference, indicating which they think is best to worst (or if the same)]*

2. Can you tell me the reasons for your answer?

3. What changes to this feedback document would make this more useful to you?

a. Prompts: What would you like to see changed about the feedback concerning prescribing errors?

b. Prompts: Do you identify any problems with this feedback report? If you do, which problems?

c. Prompts: What do you like about the presentation style?

74

d. Prompts: Is the information presented clear to you? Is the text big enough?

For peer review only

Do you like the length of the feedback document or do you think we need to add/remove some information?

4. If you want to receive feedback, how often do you want to receive a document like these?

a. Prompts: Would you prefer to receive a feedback document weekly or monthly or other?

PART 2

Thank you, now I would like you to look at the lists below.

1. 1ST column - please tick the top 5 types of data you would like to receive feedback on and why.

2. 2nd column – please rank in order the denominators you would like to be used to determine the error rate. Why?

NUMERATOR – **please tick top 5**<br>Types of prescribing error (irrespective of actual or potential patient harm)	DENOMINATOR – **please rank (1 = most preferred info, 7 = least preferred info)**
☐ Allergy<br>☐ Omission of medication<br>☐ Unnecessary prescribing<br>☐ Clinical contraindications<br>☐ Dosing errors<br>☐ Formulation errors<br>☐ Wrong route errors<br>☐ Lack of clear directions for administration<br>☐ Technical errors (e.g. no max dose)<br>☐ Duration of treatment errors<br>☐ Miscellaneous<br>☐ Other (please state):	☐ No. of NEW medications prescribed (any time)<br>☐ No. of NEW medications prescribed 9am – 5pm<br>☐ No. of NEW medications prescribed out of hours<br>☐ No. of NEW medications prescribed (by day of week)<br>☐ No. of patients under the doctor's care (by consultant)<br>☐ No. of patients in which the doctor had prescribed at least one drug (irrespective of consultant/specialty/ward)<br>☐ Other (please state):

Did you receive feedback in the past via a different way than the methods discussed in this interview?

Is there anything else you would like to add?

Thank you for participating on this interview. Your contribution is very much appreciated.

For peer review only

Example 1

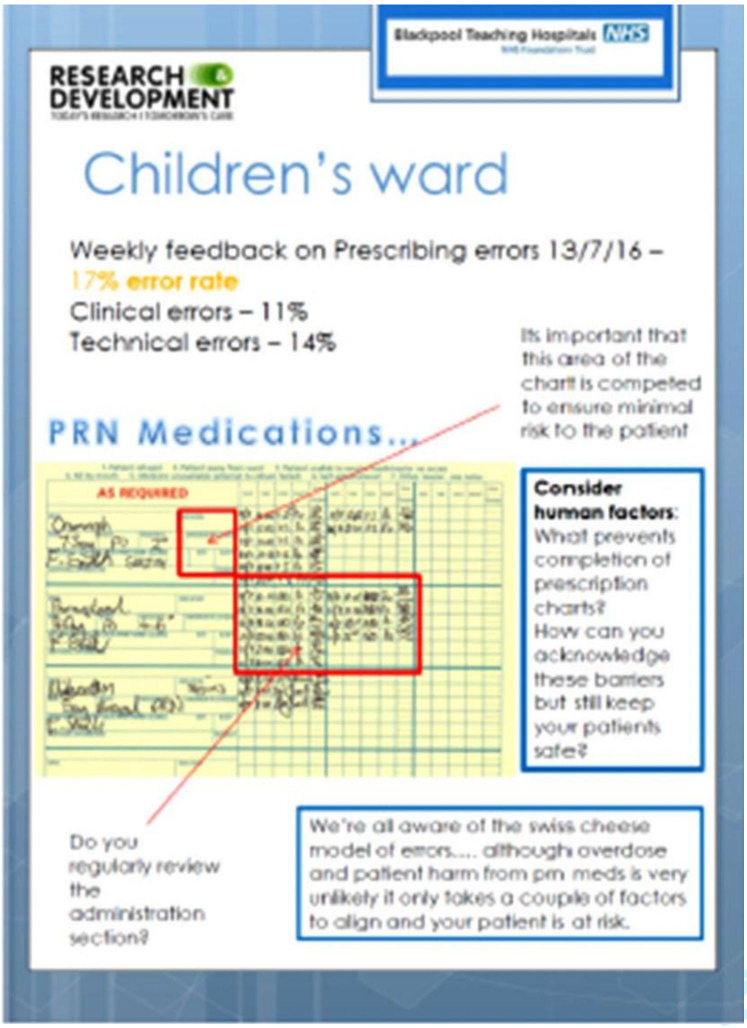
Blackpool Teaching Hospitals NHS
RESEARCH DEVELOPMENT
Children's ward
Weekly feedback on Prescribing errors 13/7/16 –
17% error rate
Clinical errors – 11%
Technical errors – 14%
Its important that this area of the chart is competed to ensure minimal risk to the patient
PRN Medications...
AS REQUIRED
Consider human factors: What prevents completion of prescription charts? How can you acknowledge these barriers but still keep your patients safe?
Do you regularly review the administration section?
We're all aware of the swiss cheese model of errors.... although overdose and patient harm from prn meds is very unlikely it only takes a couple of factors to align and your patient is at risk.

Example 2

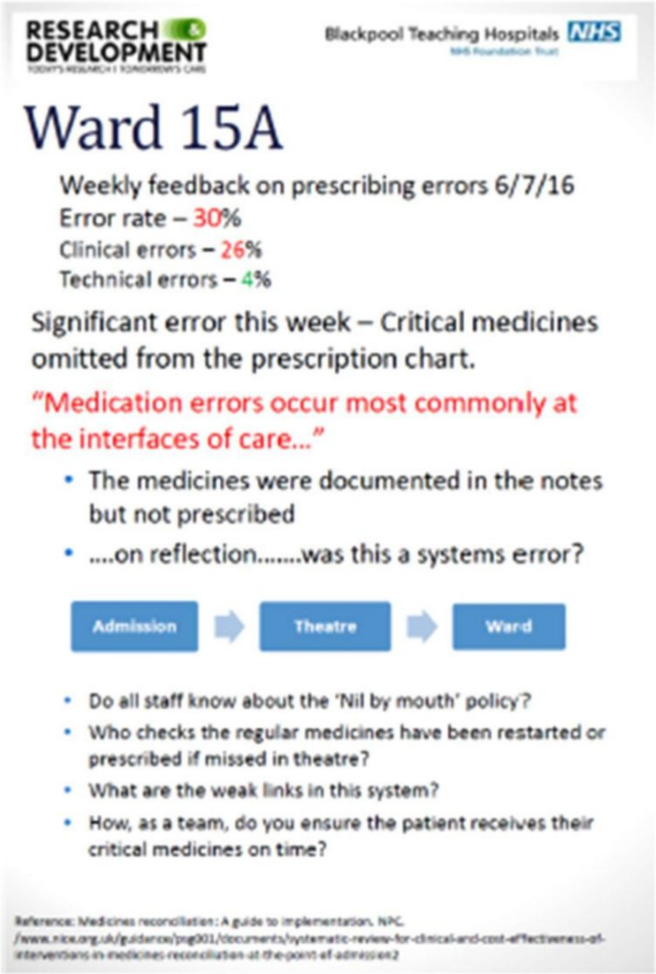

ew only

Example 3

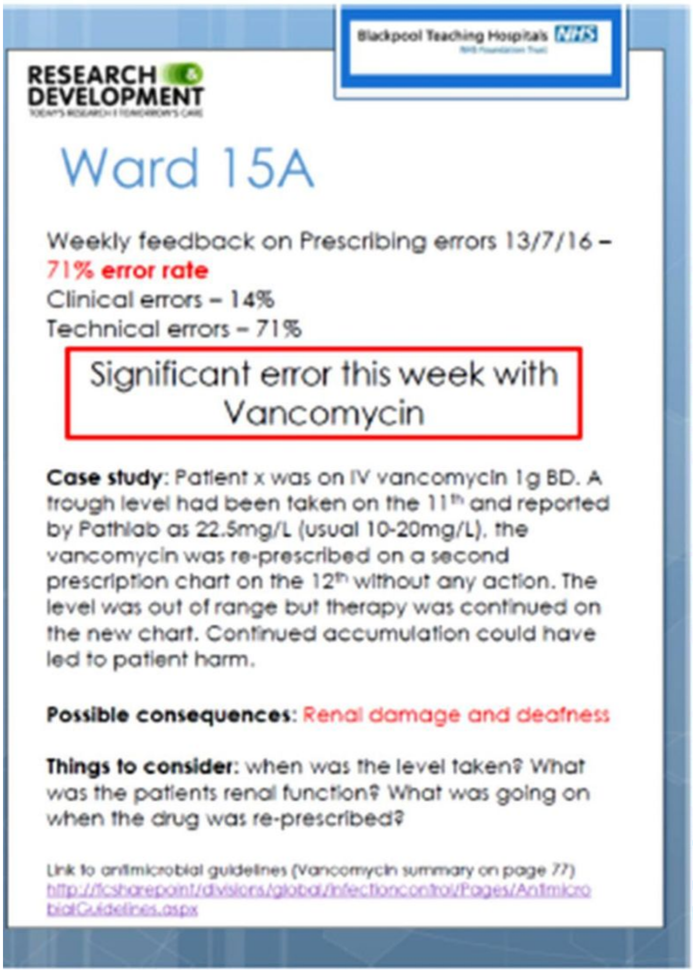
Blackpool Teaching Hospitals NHS
RESEARCH & DEVELOPMENT
Ward 15A
Weekly feedback on Prescribing errors 13/7/16 –
71% error rate
Clinical errors – 14%
Technical errors – 71%
Significant error this week with Vancomycin
Case study: Patient x was on IV vancomycin 1g BD. A trough level had been taken on the 11th and reported by Pathlab as 22.5mg/L (usual 10-20mg/L), the vancomycin was re-prescribed on a second prescription chart on the 12th without any action. The level was out of range but therapy was continued on the new chart. Continued accumulation could have led to patient harm.
Possible consequences: Renal damage and deafness
Things to consider: when was the level taken? What was the patients renal function? What was going on when the drug was re-prescribed?
Link to antimicrobial guidelines (Vancomycin summary on page 77)
http://fcsharepoint/divisions/global/infectioncontrol/Pages/AntimicrobialGuidelines.aspx

Example 4

Western Sussex Hospitals Standard Process for Routine Ward Round
Considerative Checklist for Complete Patient Review

The purpose of this process is to ensure that the team "does it all" for all the patients during the round
Make one member of the team the "Safety Checker" who uses this checklist before leaving each patient.
The checker must highlight anything omitted, speak up and get it done!
Key = [yellow] **these sections must be checked in all patients**, tick white boxes only when indicated

Date	Checker's Name	Checker's Status	Signed	Clinical Team	Type of Round
…/…./2015					**Routine**
Start time	Finish time	Number of Doctors	**Total number of patients**		
			**New**	**Review**	**No of wards**

**Aspect of Care**										
**Patient Initials**										
**Bed number**										
**Preparatory Discussions**	**Preparation Before Going to the Bedside**									
**Review Diagnoses**										
**Checked New Results**										
Clinical Thinking										
**Clinical Frailty?**										
**Report from Nurse?**										
**Consultation**	**Bedside Patient Consultation**									
**Nurse present?**										
**Hand hygiene?**										
**Introductions?**										
**Wristband check?**										
**Ask and Listen?**										
**Charts**	**Check All Relevant Bedside Charts**									
**Write the NEWS Score**										
**Drugs Chart checked?**										
On iv Fluid? Review chart										
Diabetic? Glucose levels										
**Planning**	**Decide on blood tests, radiology, Discuss Discharge Date, Ceiling of Care**									
Agree future tests?										
EDD discussed?										
MRFD?										
**7 Point Safety Check**	**7 Point Safety Check to Reduce Avoidable Harm**									
Pain or discomfort										
Eating and Drinking										
Bowel Function										
Urine / catheter?										
Cannula and iv lines										
Pressure area care										
Change VTE Rx?										
**All 7 points checked?**										
**Documentation**	**Consultant Inspects and Counter Signs Today's Notes**									
**Notes signed by Consultant?**										
**Summed Up to Patient?**										
**Reported Plan to Nurse?**										

© Dr G Caldwell January 2011

H:_1_Gordon_Assessment_and_Feedback_1Ward Round Pack_SOP Routine Ward Round 21150217.docx

Example 5

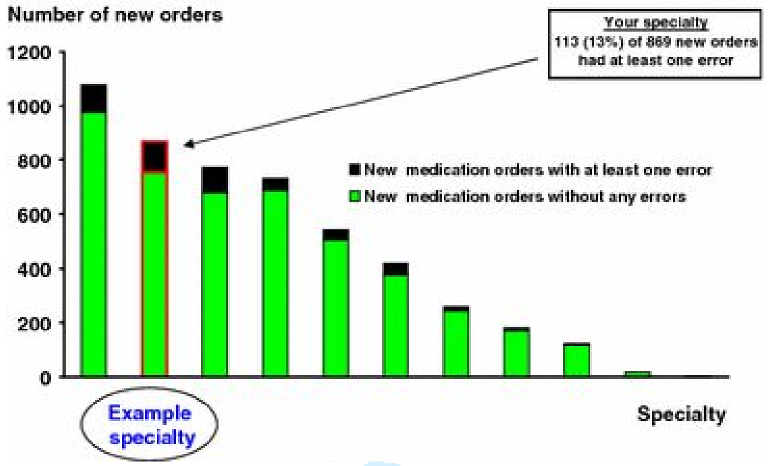
Number of new orders
Your specialty
113 (13%) of 869 new orders had at least one error
New medication orders with at least one error
New medication orders without any errors
Example specialty
Specialty

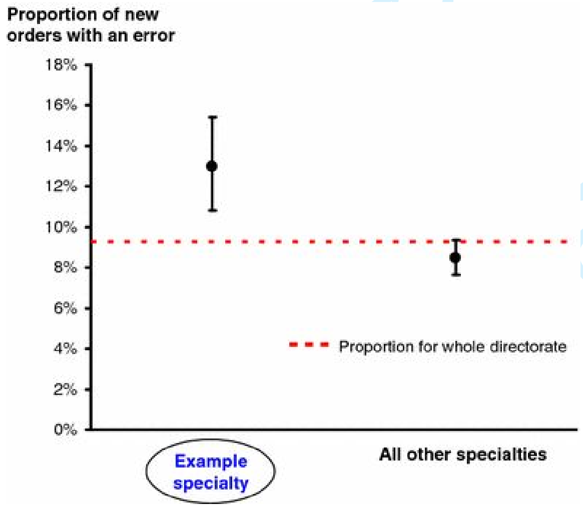
Proportion of new orders with an error
Proportion for whole directorate
Example specialty
All other specialties

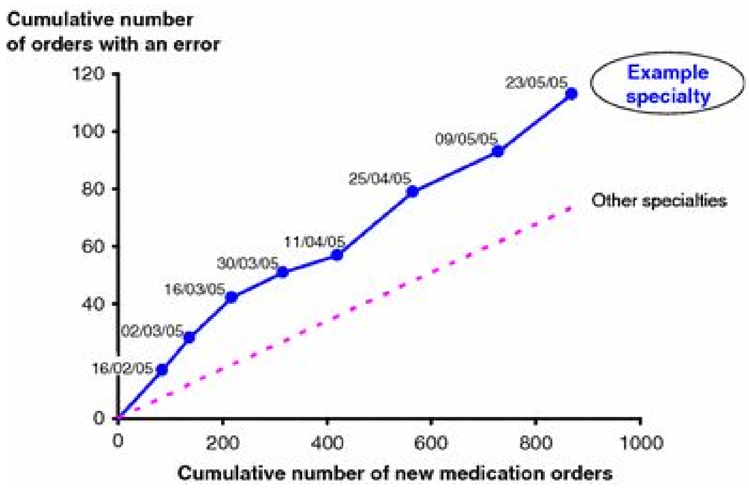
Cumulative number of orders with an error
Example specialty
Other specialties
Cumulative number of new medication orders

Example 6

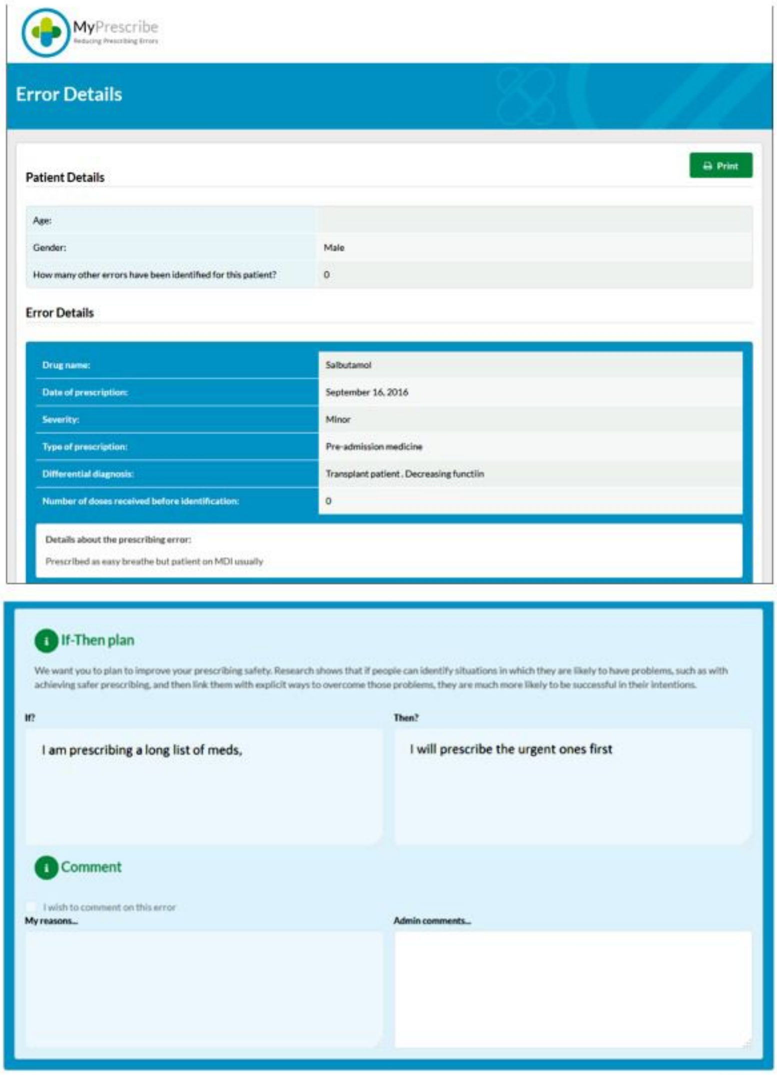

nly

BMJ Open

Learning from electronic prescribing errors: a mixed methods study of junior doctors' perceptions of training and individualised feedback data

Journal:	*BMJ Open*
Manuscript ID	bmjopen-2021-056221.R1
Article Type:	Original research
Date Submitted by the Author:	18-Aug-2022
Complete List of Authors:	Chu, Ann; Imperial College London, Faculty Education Office<br>Kumar, Arika; Imperial College Healthcare NHS Trust<br>Depoorter, Geraldine; Imperial College Healthcare NHS Trust<br>Franklin, Bryony; Imperial College Healthcare NHS Trust<br>McLeod, Monsey; Imperial College Healthcare NHS Trust
**Primary Subject Heading**:	Medical education and training
Secondary Subject Heading:	Health informatics, Pharmacology and therapeutics, Qualitative research
Keywords:	MEDICAL EDUCATION & TRAINING, GENERAL MEDICINE (see Internal Medicine), Adverse events < THERAPEUTICS, Health informatics < BIOTECHNOLOGY & BIOINFORMATICS, Quality in health care < HEALTH SERVICES ADMINISTRATION & MANAGEMENT

SCHOLARONE™
Manuscripts

I, the Submitting Author has the right to grant and does grant on behalf of all authors of the Work (as defined in the below author licence), an exclusive licence and/or a non-exclusive licence for contributions from authors who are: i) UK Crown employees; ii) where BMJ has agreed a CC-BY licence shall apply, and/or iii) in accordance with the terms applicable for US Federal Government officers or employees acting as part of their official duties; on a worldwide, perpetual, irrevocable, royalty-free basis to BMJ Publishing Group Ltd ("BMJ") its licensees and where the relevant Journal is co-owned by BMJ to the co-owners of the Journal, to publish the Work in this journal and any other BMJ products and to exploit all rights, as set out in our licence.

The Submitting Author accepts and understands that any supply made under these terms is made by BMJ to the Submitting Author unless you are acting as an employee on behalf of your employer or a postgraduate student of an affiliated institution which is paying any applicable article publishing charge ("APC") for Open Access articles. Where the Submitting Author wishes to make the Work available on an Open Access basis (and intends to pay the relevant APC), the terms of reuse of such Open Access shall be governed by a Creative Commons licence – details of these licences and which Creative Commons licence will apply to this Work are set out in our licence referred to above.

Other than as permitted in any relevant BMJ Author's Self Archiving Policies, I confirm this Work has not been accepted for publication elsewhere, is not being considered for publication elsewhere and does not duplicate material already published. I confirm all authors consent to publication of this Work and authorise the granting of this licence.

For peer review only

Title Page:

Learning from electronic prescribing errors: a mixed methods study of junior doctors' perceptions of training and individualised feedback data

Ann Chu[1], Arika Kumar[2], Géraldine Depoorter[2], Bryony Dean Franklin[2], Monsey McLeod[2]

Author affiliations:

1. **Faculty of Medicine, Imperial College London**
2. **Centre for Medication Safety and Service Quality, Imperial College Healthcare NHS Trust**

Corresponding author:

Dr Ann Chu, Academic Clinical Fellow, Faculty Education Office, Reynolds Building, St. Dunstan's Road, London W6 8RP

Email: annchu@doctors.org.uk

Abstract

Objectives: To explore the views of junior doctors towards (i) electronic prescribing (EP) training and feedback, (ii) readiness for receiving individualised feedback data about EP errors, and (iii) preferences for receiving and learning from EP feedback.

Design: Explanatory sequential mixed methods study comprising quantitative survey (phase 1), followed by interviews and focus group discussions (phase 2).

Setting: Three acute hospitals of a large English National Health Service organisation.

Participants: 25 of 89 foundation year 1 and 2 doctors completed the phase 1 survey; five participated in semi-structured interviews and seven in a focus group in phase 2.

Results: Foundation doctors in this mixed-methods study reported that current feedback provision on EP errors was lacking or informal, and that existing EP training and resources were underutilised. They believed feedback about prescribing errors to be important and were keen to receive real-time, individualised EP feedback data. Feedback needed to be in manageable amounts, motivational and clearly signposting how to learn or improve. Participants wanted feedback and better training on the EP system to prevent repeating errors. In addition to individualised EP error data, they were positive about learning from general prescribing errors and aggregated EP data. However, there was lack of consensus about how best to learn from statistical data. Potential limitations identified by participants included concern about how the data would be collected and whether it would be truly reflective of their performance.

Conclusions: Junior doctors would value feedback on their prescribing, and are keen to learn from EP errors, develop their clinical prescribing skills and use the EP interface effectively. We identified preferences for EP technology to enable provision of real-time data in combination with feedback to support learning and potentially reduce prescribing errors.

For peer review only

Article summary: Strengths and limitations of this study

- A key strength is the sequential explanatory mixed methods approach that allowed further exploration of survey responses with in-depth qualitative data from interviews and focus group.
- All foundation doctors in the organisation were invited to participate; this enabled capture of a range of views and experiences of electronic prescribing (EP) training and feedback that could be used to identify targets for improvement.
- Findings from a single organisation may not be generalisable to other hospital settings, but may be useful for those using similar EP systems and providing similar types of training.

For peer review only

Word count 4376

Background

Prescribing errors are reported to affect 7% of inpatient medication orders (1). Worldwide, medication errors comprise a global patient safety issue, costing an estimated £33 billion (2).

Electronic prescribing (EP) is generally advocated for reducing prescribing errors but studies have typically focussed on the technology and not the human factors or educational aspects (3). A systematic review highlighted that EP systems can introduce other error types, including incomplete computerised displays, selection errors in drop-down menus, reliance on default settings and overdependence on clinical decision support (4). Therefore, the transition to electronic prescribing (EP) in the English National Health Service (NHS) introduces an additional component for which staff require training.

Medication safety research based on theories of human error suggests that prescribing errors occur as a result of latent and environmental conditions aligning with, or contributing to, failures in defences (5). Learning from prescribing errors requires an understanding of the context (both at system and individual level) and that feedback be provided for relevant stakeholders, including prescribers. Past research has generally focused on measuring prescribing errors or root cause analysis (6–10); more recent publications on feedback interventions to improve prescribing are still primarily in the context of paper drug charts. (11–14)

In the UK, newly qualified ('junior') doctors have previously been identified as having one of the highest error rates among prescribers, as well as doing most inpatient prescribing (6,15). The UK Foundation Programme is a 2-year mandatory training programme for newly qualified medical graduates. The Royal College of Physicians recognises that supporting junior doctors in safe prescribing requires education, practical resources and a culture of patient safety (16). Numerous studies have identified this novice prescriber group as a focus for educational interventions but to date, most have been in the context of paper-based prescribing(3,11–14).

A previous questionnaire study of junior doctors and hospital pharmacists identified that receiving structured feedback, including prescribing rates, was well received (17). However, this work was conducted in the context of paper-based prescribing and required resource-intensive manual audits; this resulted in considerable delay for feedback and was not sustainable for routine implementation. We have therefore identified a literature gap in relation to EP feedback for junior doctors. This study aimed to explore the views of junior doctors towards (i) the EP training and feedback they receive, (ii) readiness for receiving individualised EP feedback data and (iii) preferences for how they would like to receive and learn from EP feedback.

Methods

Setting

We conducted a mixed-methods study at three large hospitals within an English NHS organisation (trust). The organisation has approximately 10,000 staff serving a population of one million people. EP has been active in most inpatient areas since 2016. This study focussed on one organisation so that participants were referring to the same EP system and had equal access to training resources.

Classroom-based training was offered at trust induction for junior doctors. Optional EP training resources include online learning modules (generic and specialty-specific), 'Quick Reference Guides' on the trust's intranet, some postgraduate teaching sessions, and EP technical support. EP 'order sentences' with default doses were available for many commonly used medications; clinical decision support tools such as allergy and drug interaction checking were not in routine use at the time of the study.

Participants

All foundation doctors in their first two years of practice [Foundation Years 1 (FY1) and 2 (FY2)] at the trust (n=89) were invited to participate. Participant invitations were circulated by the Trust postgraduate education team and blinded to the research team. The year groups were approximately equal in size.

Study design

This was an explanatory sequential mixed methods study comprising two phases: phase 1 was a quantitative questionnaire delivered to all foundation doctors in February/March 2018; phase 2 was a qualitative interview and focus group study with volunteer participants from the same population in April/May 2018. This study design was chosen to permit analysis of the quantitative data for broad perspectives followed by in-depth exploration of themes that required further explanation.

Ethics

This study met the Health Research Authority criteria for service evaluation; NHS ethics approval was not required. It was registered as a service evaluation within the trust (reference 266). The study was conducted in line with ethical research practice: ensuring voluntary participation, obtaining written consent for interviews and focus groups, and anonymising data in line with data protection regulations.

Patient and public involvement

There was no patient and public involvement in this study.

Data collection

For peer review only

Phase 1 questionnaires were distributed online and on paper. Email circulation via postgraduate education teams ensured that all foundation doctors working in the organisation had an opportunity to participate. The email summarised the purpose of the study and included a weblink to an online version of the questionnaire. In addition, paper questionnaires were distributed at teaching sessions.

In phase 2, the same population of foundation doctors were invited to participate in interviews. In addition, a lunchtime focus group was offered to maximise recruitment. Due to the anonymous nature of the survey, we did not confirm if any of the participants contributed to both phases. However, we decided that maximising recruitment for phase 2 was essential and invited the whole population to participate.

Phase 1 – questionnaire study

The questionnaire comprised 65 questions (Appendix 1). This was based on a questionnaire used in a previous study (17). There were two major adaptations: (i) questions were adapted to reference EP (rather than paper-based prescribing), and (ii) clinical scenarios were added. Most responses required were on five-point Likert scales ranging from 'strongly disagree' to 'strongly agree'. Where relevant, there were additional options for 'not used' or 'not available'. The questionnaire was not psychometrically tested.

Four clinical scenarios were devised by research pharmacists as examples of prescribing errors with different levels of harm. These were based on categories from a national incident reporting system comprising 'no harm', 'low harm', 'moderate harm' to 'severe harm' (18) .The fifth category of 'death' was not included. Clinical details were devised from clinical experience and knowledge of local prescribing governance committees. The scenarios were pre-piloted by four pharmacists and one doctor for face and content validity.

Phase 2 – qualitative interview and focus group study

Interviews and a focus group were conducted using a pre-piloted topic guide (Appendix 2). This was formulated after an interim analysis of survey results in phase 1 and identified areas of further interest. Six example prescribing error feedback formats were obtained from the published literature and shown to participants. A table of EP error types (e.g. allergies, dosing errors) was also used as a prompt for discussion. Handwritten notes were recorded for both the focus group and the interviews, which included some verbatim comments from participants.

Data analysis

For peer review only

Phase 1 quantitative data were summarised using descriptive statistics, using Microsoft Excel. Positive responses ('agree and strongly agree') and negative responses ('disagree and strongly disagree') were combined when reporting the data. Neutral responses were included in figures 1-3.

Phase 2 interview and focus group notes were transcribed and then analysed thematically using an inductive approach. This involved systematic coding of the transcripts, organising codes into categories followed by grouping into broader themes (Appendix 3). Two researchers conducted this independently, with quality assurance by a third, more experienced researcher. Comparison between the interview and focus group data sets suggested that themes were similar and could be integrated. Themes were discussed to consensus. Member checking was not used.

The quantitative and qualitative data were then further integrated at the discussion phase. This included reflection on whether the data was complimentary or if there were areas of divergence. This was discussed to consensus by the wider research team.(19,20)

For peer review only

Results

In the phase 1 quantitative study, 25 of 89 (28%) foundation doctors submitted responses. Respondents comprised 14 females (56%), 8 males (32%) and 3 whom did not disclose their gender. The majority were FY1 (n=16), six were FY2 (n=6) and three did not indicate their grade. Respondents were from 14 different medical schools.

In the phase 2 qualitative study, 12 participants took part in either a focus group (n=7) or an individual interview (n=5). Eight were FY1 and four were FY2.

Phase 1: Quantitative survey data

Current level of EP training and feedback

Foundation doctors reported varied experiences of EP and associated training resources [figure 1]. They were equivocal as to whether EP reduced the number of prescribing errors that led to patient harm (12/25 agreed). Similarly, about half were satisfied with the training that had been provided at induction (13/25). More than half were satisfied with the availability of resources to enable them to prescribe safely (18/25). However, there was lack of awareness and/or use of specifically tailored training resources. Some reported not having received EP classroom training (7/25) and 13/25 reported not using the specialty-specific training modules. The majority reported not receiving the training provided as part of the postgraduate education sessions (14/25) or not having used the 'Quick Reference Guides' and crib sheets available on the intranet (17/25). However, in free text responses, respondents perceived that training on the EP interface would be useful, and that better understanding of the EP system might help prevent errors.

The majority of respondents said they were not always informed of their own prescribing errors (17/25) and that this was of concern. If they did receive feedback, this was generally in an unplanned or unscheduled way (21/25). Free text responses reconfirmed that "little" or "rare" feedback was received. Junior doctors stated they would seek EP help from their peers (23/25), pharmacists (22/25) or more senior doctors (12/25). However, they said they generally did not ask nurses (6/25) or consultants (2/25).

Readiness for receiving individualised EP feedback data

Most respondents (23/25) believed receiving feedback about prescribing errors was important and that being aware of their own errors would help improve their prescribing [figure 2]. They believed receiving formal feedback on prescribing errors would reduce future errors (22/25). Foundation doctors wanted to know about all prescribing errors they made (22/25), including minor errors (19/25) and confirmed this when presented with example clinical scenarios covering 'no harm' (18/25) and 'low harm' (22/25).

Preferences for receiving and learning from individualised EP feedback

Participants were keen for individualised feedback data to be timely; they supported a variety of methods including verbal (19/25), instant messaging (22/25) and/or EP system emails (18/25) [Figure 3].

For peer review only

Respondents were receptive to the idea of receiving a regular report (20/25) and the ability to track errors made over time (19/25). They were keen to review personal EP errors with a pharmacist (19/25). However, there was lack of consensus about doing this with their peers (7/25 agreed, 9/25 disagreed) and opposition to reviewing EP errors with a consultant (6/25 agreed; 13/25 disagreed).

Participants were also keen to learn from others' prescribing errors that were relevant to their practice (20/25). This information could be received in several ways, including on the ward (14/25), in existing training sessions (17/25) or new dedicated sessions (13/25). They were also receptive to receiving a regular summary of common prescribing errors within their specialty (20/25), rare but serious errors in their specialty (18/25), and in the organisation (15/25).

Phase 2: Qualitative interview and focus group data

Preferences for receiving and learning from EP feedback

Participants discussed both general prescribing feedback and their perceptions of EP-specific feedback. Participants' preferences for receiving and learning from prescribing feedback were grouped into four themes: **(i) nature of individualised feedback data, (ii) context of feedback, (iii) learning from feedback, and (iv) EP-specific feedback**.

(i) Nature of individualised feedback data

The most consistent theme among participants was the need for timely feedback when specific errors were detected. This was especially important for significant clinical errors. Due to working patterns, the original prescriber might not be aware of their prescribing error at all. Feedback on a specific error within a week or two was required to optimise recall of the patient's clinical situation and the doctor's working context. More timely feedback using the EP system was a perceived advantage expressed by participants, compared to feedback based on paper drug charts and manual audit.

Participants wanted individualised feedback data to be specific, tailored and concise. They also focussed on the learning aspect rather than the error itself and wanted feedback to be non-judgemental, balanced and motivational, especially if delivered by email.

"It would be nice to have a headline that says something like 'congratulations, you only made two errors this past month' – give some positive feedback as well as information about prescribing error" (FY2, Interview 2).

"Error rate sounds a bit judgey [sic] and feels like an attack, feels like you've been watched" (FY1, Focus group)

In terms of format and content, there was no consensus on preferences around the example reports shared. Instead, a combination of text, illustrations, graphs and case studies were generally considered to be useful.

Context of feedback

Although participants were generally positive about receiving feedback on EP errors, this depended on the context in which it was given and received. An important factor was the feedback provider. EP feedback in a report from an anonymised source was perceived to be potentially problematic due to workload pressures and limited time to focus on emails. Most participants had received verbal feedback from their ward pharmacist who had corrected an error and were comfortable with this if they had worked with them before. There was a degree of trust and confidence in particular with specialist clinical pharmacists (e.g. renal, paediatrics) as they were perceived to understand the nuances of prescribing for their patient cohort and were able to better highlight clinically relevant errors. However, participants raised concern about the potential workload for their ward pharmacist if more regular formal feedback was to be delivered due to the time required for data collection and also being able to reciprocate with time to receive feedback.

"Sometimes pharmacists will say "oh you do know that you got this wrong" but it's done casually" (FY2, interview 1).

There was consensus from participants that feedback on more significant prescribing errors (i.e. those associated with potential or actual 'major harm') was very important. The value of feedback for less serious errors was less convincing as participants perceived that they would not have time to focus on all errors. When given a list of example error types, participants ranked prescribing a drug to which a patient has an allergy as the most important error to receive feedback on. Clinical contraindications, omissions and unnecessary prescribing were also prioritised for feedback. However, dosing errors and formulation errors were not.

(ii) Learning from feedback

Participants acknowledged that learning from prescribing feedback could take place at an individual level, in a group setting or even at organisational level.

There were mixed feelings about learning from data such as baseline error rates; some participants perceived this to be helpful for context, whilst others felt that statistics (especially in isolation) would not be as helpful for learning in comparison to narrative feedback. Similarly, benchmarking statistics with peers had a mixed response; some participants valued the quantitative aspect while others were unclear about how this would advance their learning and were afraid of being judged.

"Error rates is like a percent on your exams…It doesn't say anything, and you can't learn from it" (FY1, focus group)

However, there was general agreement that learning about prescribing errors in the form of case studies was a helpful educational tool. Learning about errors made by peers in a group setting was useful, especially as foundation doctors rotated specialties. However, it was noted that reporting on real EP errors publicly needed to be done carefully and anonymously, as there was concern about having a negative impact: *"…it might break the team spirit" (FY1, focus group).*

One participant was clear that all prescribing errors were significant for patient safety and that accepting minor errors was suggestive of professional 'laziness'. Instead, they felt that the organisation should be learning from all types of errors and cultivating good prescribing practice:

"We as a hospital are lazy… for example, in giving the indication for medications" (FY2, interview 4).

The team prescribing culture was felt to be important, as junior doctors were influenced by senior decision makers, particularly in terms of bad habits or prescribing outside the protocol. Moreover, prescribing decisions were often made by the consultant but enacted by the junior doctors.

For peer review only

Therefore, one participant suggested providing feedback to the team in general as well as individuals.

"The junior doctors are a lot influenced by the senior doctors and there is a hierarchy, sometimes I want to say something… but it's in some way difficult to correct them. But anyway, I just say it… because patient safety is the priority… It's the job of the entire team to look at the drug chart and review it" (FY1, interview 4).

In addition, error rates on its own might not be helpful for comparing between specialties with different prescribing cultures, therefore reducing the validity of the data.

"Graphs are a nice idea in some ways… but if you are in a specialty and you are not making many errors, it's weird to be like oh well, I do way better than the other specialties… how serious are these errors on the graphs? If you do badly it's embarrassing for the team, but it can be a good thing for some… maybe they are going to do more to do their best" (FY2, interview 5)

(iii) EP-specific feedback

Participants perceived that the EP system architecture was capable of providing greater prescribing support than paper prescribing and there was a potential for EP data to be used for feedback. Participants wanted to have a better understanding of the EP system to avoid repeating errors and queried how EP error data would be accurately collected and judged. There was anxiety about being monitored and that automated EP data might not accurately reflect their abilities.

There were some examples of participants who had difficulties transitioning from paper-based prescribing. Specifically, participants found that the default settings for standard drug administration times made it more difficult to prescribe complex scheduling times, reschedule drug administration times, and suspend medication doses. These EP-specific challenges exposed them to risks of making EP errors. Participants were keen to know how to use the EP system better in these situations and how the system could be adapted to be more intuitive.

Some participants also suggested that the national student prescribing safety assessment was not a good marker for preparedness and was not helpful for EP skills: *"it's very different from actually using the system" (FY2, interview 5).*

Discussion

Key findings

Foundation doctors in this mixed-methods study reported that current feedback provision on EP errors was lacking or informal, and that existing EP training and resources were underutilised. This feedback gap has been previously identified (17)and persists despite quality improvement initiatives at the study organisation. However, these have been difficult to sustain due to the use of manual audit and human resources, including individual ward pharmacists (21,22) Other studies have also identified that provision of safe prescribing training and induction is variable (including e-learning) due to the lack of a standardised prescribing curriculum for postgraduate doctors (23,24) .

Survey participants believed feedback about prescribing errors to be important and indicated readiness to receive real-time, individualised EP feedback data. Participants in the interviews and focus group expanded that feedback also needed to be in manageable amounts, motivational and

clearly signposting how to learn or improve. Survey participants wanted feedback and better EP training to prevent repeating errors. In addition to individualised EP error data, they were receptive to learning from general prescribing errors and aggregated EP data. However, focus group and interview participants revealed that despite this surface acknowledgement, there was lack of consensus about how best to learn from statistical data. Potential limitations included concern about how the data would be collected, whether it reflected clinical ability, and time to reflect. Concerns about being performance managed has also been highlighted in other patient safety research with junior doctors (25).

Participants in the survey, interviews and focus group all identified ward pharmacists as a trusted source and provider of feedback. This is consistent with other studies evaluating pharmacist-led feedback interventions (11,12,14,26).

Interpretation

This study supports the findings of previous research that foundation doctors want feedback on prescribing errors (17). There is hope that EP technology could facilitate feedback data in a timely, individualised and digitised format to optimise learning. However, recent initiatives to extract EP data for this purpose is still challenging (27).In addition, provision of EP error data in isolation would not be adequate to promote reflective practice and prevent further EP errors. This is consistent with other research suggesting that email feedback alone is less effective than group discussions or directly observed prescribing encounters(28). In our study, participants described contextualising EP errors using illustrative case studies and having the opportunity to debrief with a pharmacist as potential learning opportunities. Enabling novice prescribers to have enough time and cognitive space to both consider the feedback and formulate an action plan were identified as potential challenges. An electronic feedback tool involving individualised learning plans has been described by others (29).

Participants did not agree on the utility of statistical data as a feedback mechanism. However, a Cochrane review suggests that audit and feedback interventions in healthcare are an effective method of stimulating behaviour change (30). Perceptual control theory also recognises the role of 'benchmarking' against a 'reference standard' as a key motivator for behaviour change (28). This might be due to the study exploring doctors' perceptions rather than a direct evaluation of experience.

In addition, interview participants discussed 'feedback', but it was not always clear whether they were distinguishing between general prescribing feedback and provision of EP data. It is therefore possible that participants and researchers were discussing different aspects of 'feedback' but using the term interchangeably to mean different things. This has also been found in the educational literature, where there is well-recognised incongruence between learners and teachers as to the definition, quantity and quality of feedback given and received (31).

Strengths and limitations

The main strength of this study came from combining quantitative and qualitative data collection methods. This gave us the opportunity to explore the research question from different perspectives. The survey was completed by more participants and captured a broader range of respondents, while the interviews and focus group gave us opportunity to probe responses in more depth. Eliciting the challenges perceived by foundation doctors was a strength of the qualitative data, as there were

For peer review only

some underlying anxieties that were not readily captured by the survey data. The multidisciplinary nature of the research team (doctor and pharmacists, with medical education and medication safety expertise) was also a strength as we were able to contribute different perspectives to the data analysis from the context of our professional experience.

Limitations of this study include the low response rate and small sample size from a single organisation, which limits generalisability. It was not feasible to expand recruitment to other institutions or extend the timeline of the study. However, themes were being repeated, suggesting that the data was approaching saturation. There are differences between FY1 (newly qualified) and FY2 (independently registered) doctors, so including both cohorts in this study population may make it more difficult to design tailored interventions. The questionnaire was not psychometrically tested. There may be participation bias, particularly with the interviews and focus group, as respondents may be more likely to be interested in the study concept. They may also have provided socially desirable responses rather than their true viewpoints. We explored participants' perceptions rather than conducting a direct evaluation in practice. Participants therefore made hypothetical assumptions about the capability of the EP system and provision of EP data. The examples that participants were shown in the interviews were based on the existing literature at the time, and thus were mainly paper-based.

Implications for practice

This data informs future work for developing individualised, digital EP feedback data for prescribers. Feedback needs to be timely, concise and relevant, with a blended educational approach in terms of providing data and example case studies, both verbally and electronically. Situational factors should be acknowledged, and feedback should be supportive and non-judgemental. Consideration should be given to the role of pharmacists in providing feedback, allocating sufficient time for prescribers to focus on feedback, prioritising the relative importance of EP errors and support formulation of action plans. Other learning points include improving awareness and engagement of EP training opportunities, changing the focus to prescribing accuracy (rather than on errors), maximising opportunity for reflective practice (not just providing information) and enabling individuals to contribute to organisational learning and improvements (for example, reporting EP system defaults). A challenge to achieving this in practice includes staffing and time resource, as well as digital capability.

Future research

Future research should focus on the evaluation of EP feedback mechanisms and integrating this into clinical practice for novice prescribers. The feasibility, acceptability and utility of these tools should be considered and appropriate mechanisms for providing context and promoting learning put into place. Challenges, unintended consequences and resource implications of introducing a novel EP feedback interface should also be captured. Our study focused on junior doctors as the main prescriber in hospitals, but in the UK, independent nurse and pharmacist prescribers also have an important role, thus provision of prescribing feedback to multiprofessional prescribers should be explored. Future educational interventions could consider combining individualised EP feedback data with modern initiatives including simulation and video-stimulated feedback (13,32).

For peer review only

Conclusion

The digital transformation of hospital inpatient prescribing presents a timely opportunity to develop, implement and evaluate an individualised prescribing feedback and learning initiative to reduce prescribing errors. Foundation doctors in this study are receptive to receiving value feedback on their prescribing, keen to learn from errors to develop their clinical prescribing skills and want to use the electronic interface effectively.

Our findings suggest a range of educational options would be a positive strategy to enhance the value of individualised EP feedback data for novice prescribers. We found a demand for EP technology to enable provision of real-time feedback to support learning, thus potentially reducing prescribing errors. The next steps are to develop a feasible approach for extracting EP data, providing individualised EP feedback data and evaluating its utility and effectiveness in practice.

For peer review only

List of figures:

Figure 1. Survey results: current level of electronic prescribing (EP) feedback and training reported by Foundation doctors (numbers correspond with number of respondents, total 25 participants)

Figure 2. Survey results: readiness for receiving electronic prescribing (EP) feedback reported by Foundation doctors (numbers correspond with number of respondents, total 25 participants)

Figure 3. Survey results: preferences for receiving and learning from electronic prescribing (EP) feedback reported by Foundation doctors (numbers correspond with number of respondents, total 25 participants)

For peer review only

Authors' contributions:

MM and BDF conceived the study and study design. Methods were developed by MM, BDF, GD, and AK. Data were collected by AK, GD, and MM and initial data analyses were conducted by GD, AK, AC and MM. Interpretation of the data was discussed to consensus by AC, BDF, and MM. The manuscript was prepared and revised by AC and MM. All authors reviewed and finalised the manuscript.

Competing interests statement:

AC – no competing interests

AK – no competing interests

GD – no competing interests

BDF supervised a PhD student who was part funded by Cerner (a supplier of electronic health record systems) but whose work was unrelated to this study.

MM – no competing interests

Funding statement:

This study was supported by the National Institute for Health Research (NIHR) Imperial Patient Safety Translational Research Centre (PSTRC-2016-004). AC is an NIHR funded Academic Clinical Fellow. The views expressed are those of the author(s) and not necessarily those of the NIHR or the Department of Health and Social Care.

Data sharing statement:

Data are available upon reasonable request.

Ethics approval statement:

This study met the Health Research Authority criteria for service evaluation; NHS ethics approval was not required. It was registered as a service evaluation within the trust (reference 266). The study was conducted in line with ethical research practice: ensuring voluntary participation, obtaining written consent for interviews and focus groups, and anonymising data in line with data protection regulations. There was no patient and public involvement in this research.

For peer review only

References

1. Lewis PJ, Dornan T, Taylor D, Tully MP, Wass V, Ashcroft DM. Prevalence, incidence and nature of prescribing errors in hospital inpatients: A systematic review. Drug Safety. 2009;32(5):379–89.

2. Donaldson LJ, Kelley ET, Dhingra-Kumar N, Kieny MP, Sheikh A. Medication Without Harm: WHO's Third Global Patient Safety Challenge. The Lancet. 2017;389(10080):1680–1.

3. The Health Foundation. Evidence scan: Reducing prescribing errors. 2012;(April):36.

4. Brown CL, Mulcaster HL, Triffitt KL, Sittig DF, Ash JS, Reygate K, et al. A systematic review of the types and causes of prescribing errors generated from using computerized provider order entry systems in primary and secondary care. Journal of the American Medical Informatics Association. 2017;24(2):432–40.

5. Puaar SJ, Franklin BD. Impact of an inpatient electronic prescribing system on prescribing error causation: A qualitative evaluation in an English hospital. BMJ Quality and Safety. 2018;27(7):529–38.

6. Dornan T, Ashcroft D, Heathfield H, Lewis P, Miles J, Taylor D, et al. An in-depth investigation into causes of prescribing errors by foundation trainees in relation to their medical education: EQUIP study. London; 2009.

7. Ryan C, Ross S, Davey P, Duncan EM, Francis JJ, Fielding S, et al. Prevalence and causes of prescribing errors: The PRescribing Outcomes for Trainee Doctors Engaged in Clinical Training (PROTECT) study. PLoS ONE. 2014 Jan 3;9(1).

8. McLellan L, Tully MP, Dornan T. How could undergraduate education prepare new graduates to be safer prescribers? British Journal of Clinical Pharmacology. 2012;74(4):605–13.

9. Lewis PJ, Seston E, Tully MP. Foundation year one and year two doctors' prescribing errors: A comparison of their causes. Postgraduate Medical Journal. 2018;94(1117):634–40.

10. Papoutsi C, Mattick K, Pearson M, Brennan N, Briscoe S, Wong G. Social and professional influences on antimicrobial prescribing for doctors-in-training: A realist review. Journal of Antimicrobial Chemotherapy. 2017 Sep 1;72(9):2418–30.

11. McLellan L, Dornan T, Newton P, Williams SD, Lewis P, Steinke D, et al. Pharmacist-led feedback workshops increase appropriate prescribing of antimicrobials. Journal of Antimicrobial Chemotherapy. 2016 May 1;71(5):1415–25.

12. Parker H, Farrell O, Bethune R, Hodgetts A, Mattick K. Pharmacist-led, video-stimulated feedback to reduce prescribing errors in doctors-in-training: A mixed methods evaluation. British Journal of Clinical Pharmacology [Internet]. 2019 Oct 23;85(10):2405–13. Available from: https://onlinelibrary.wiley.com/doi/10.1111/bcp.14065

13. Green W, Shahzad MW, Wood S, Martinez Martinez M, Baines A, Navid A, et al. Improving junior doctor medicine prescribing and patient safety: An intervention using personalised, structured, video-enhanced feedback and deliberate practice. British Journal of Clinical

For peer review only

Pharmacology [Internet]. 2020 Nov 18;86(11):2234–46. Available from: https://onlinelibrary.wiley.com/doi/10.1111/bcp.14325

14. Lloyd M, Watmough SD, O'Brien S v., Hardy K, Furlong N. Evaluating the impact of a pharmacist-led prescribing feedback intervention on prescribing errors in a hospital setting. Research in Social and Administrative Pharmacy. 2021 Sep 1;17(9):1579–87.

15. Ryan C, Ross S, Davey P, Duncan EM, Francis JJ, Fielding S, et al. Prevalence and causes of prescribing errors: The PRescribing Outcomes for Trainee Doctors Engaged in Clinical Training (PROTECT) study. PLoS ONE. 2014;9(1):1–9.

16. Ferner R. Supporting junior doctors in safe prescribing Supporting junior doctors in safe prescribing. Royal college of Physicians. 2017;

17. Bertels J, Almoudaris AM, Cortoos PJ, Jacklin A, Franklin BD. Feedback on prescribing errors to junior doctors: Exploring views, problems and preferred methods. International Journal of Clinical Pharmacy. 2013;35(3):332–8.

18. NHS. NRLS Organisation patient safety incident workbook. 2017;(September).

19. O'Cathain A, Murphy E, Nicholl J. Three techniques for integrating data in mixed methods studies. BMJ. 2010 Sep 17;341(sep17 1):c4587–c4587.

20. O'Cathain A, Murphy E, Nicholl J. The quality of mixed methods studies in health services research. Journal of Health Services Research and Policy. 2008 Apr;13(2):92–8.

21. Franklin BD, O'Grady K, Paschalides C, Utley M, Gallivan S, Jacklin A, et al. Providing feedback to hospital doctors about prescribing errors; a pilot study. Pharmacy World and Science. 2007 Jun;29(3):213–20.

22. Reynolds M, Jheeta S, Benn J, Sanghera I, Jacklin A, Ingle D, et al. Improving feedback on junior doctors? prescribing errors: Mixed-methods evaluation of a quality improvement project. BMJ Quality and Safety. 2017;26(3):240–7.

23. Cheetham J, Mathews R, Sanghvi S, MacKenna B, Mitchell F, Provis E, et al. What does a good prescribing induction for the newly qualified doctor look like? Future Healthcare Journal. 2020 Feb;7(Suppl 1):s88–9.

24. Kennedy MB, Malik M, Haq I, Williams SE, Okorie M. Safe prescribing training provision for junior doctors: is this optimal? BMC Medical Education [Internet]. 2016 Dec 24;16(1):220. Available from: http://bmcmededuc.biomedcentral.com/articles/10.1186/s12909-016-0748-4

25. Redwood S, Ngwenya NB, Hodson J, Ferner RE, Coleman JJ. Effects of a computerized feedback intervention on safety performance by junior doctors: Results from a randomized mixed method study. BMC Medical Informatics and Decision Making. 2013;13(1):1–10.

26. Axon DR, Lim RHM, Lewis PJ, Sandher S, Thondee J, Edwards K, et al. Junior doctors' communication with hospital pharmacists about prescribing: Findings from a qualitative interview study. European Journal of Hospital Pharmacy . 2018 Sep 1;25(5):257–61.

27. McLeod M, Farah S, Macaulay K, Sheth T, Patel M, Ghafour A, et al. Designing a continuous data-driven feedback and learning initiative to improve electronic prescribing: an interdisciplinary quality improvement study. International Journal of Pharmacy Practice. 2021 Mar 26;29(Supplement_1):i18–9.

For peer review only

28. Ferguson J, Keyworth C, Tully MP. 'If no-one stops me, I'll make the mistake again': Changing prescribing behaviours through feedback; A Perceptual Control Theory perspective'. Research in Social and Administrative Pharmacy. 2018 Mar 1;14(3):241–7.

29. Keyworth C, Hart J, Thoong H, Ferguson J, Tully M. A Technological Innovation to Reduce Prescribing Errors Based on Implementation Intentions: The Acceptability and Feasibility of MyPrescribe. JMIR Human Factors. 2017;4(3):e17.

30. Ivers N, Jamtvedt G, Flottorp S, Young JM, Odgaard-Jensen J, French SD, et al. Audit and feedback: Effects on professional practice and healthcare outcomes. Cochrane Database of Systematic Reviews. 2012;2012(6).

31. Ajjawi R, Regehr G. When I say … feedback. Medical Education. 2019;53(7):652–4.

32. Patel R, Green W, Shahzad MW, Church H, Sandars J. Using a self-regulated learning-enhanced video feedback educational intervention to improve junior doctor prescribing. Medical Teacher [Internet]. 2020 Aug 2;42(8):886–95. Available from: https://www.tandfonline.com/doi/full/10.1080/0142159X.2020.1748183

For peer review only

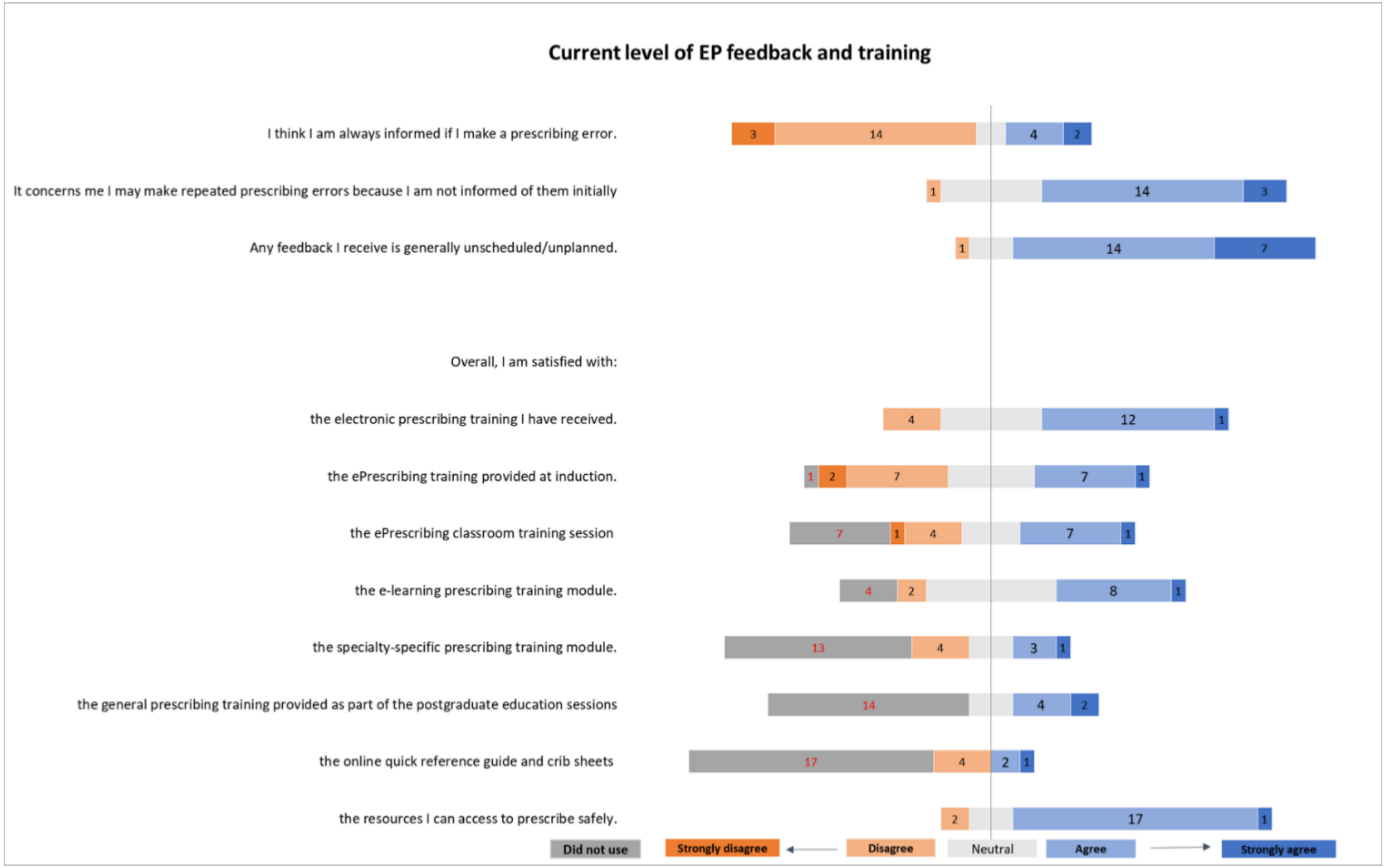

Figure 1. Survey results: current level of electronic prescribing (EP) feedback and training reported by Foundation doctors (numbers correspond with number of respondents, total 25 participants)

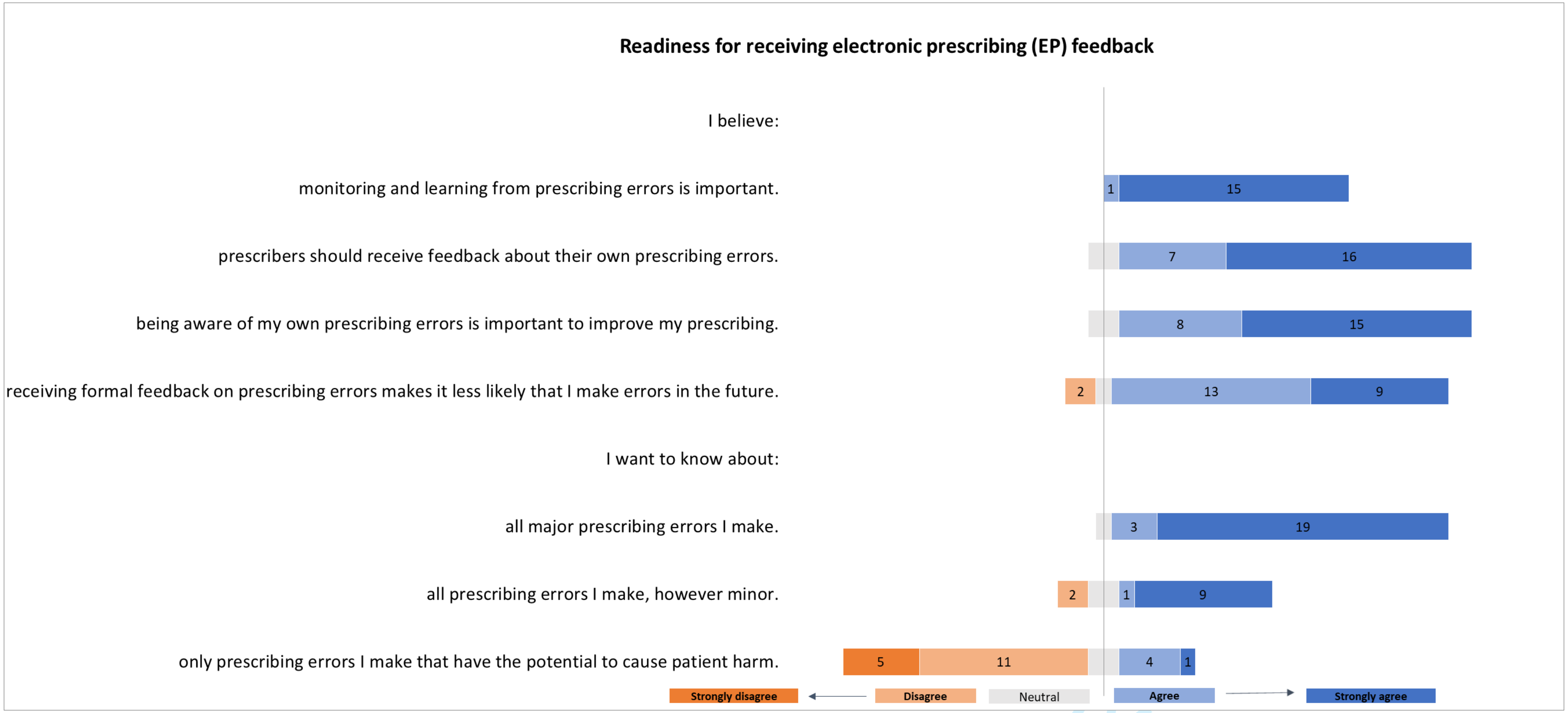

Figure 2. Survey results: readiness for receiving electronic prescribing (EP) feedback reported by Foundation doctors (numbers correspond with number of respondents, total 25 participants)

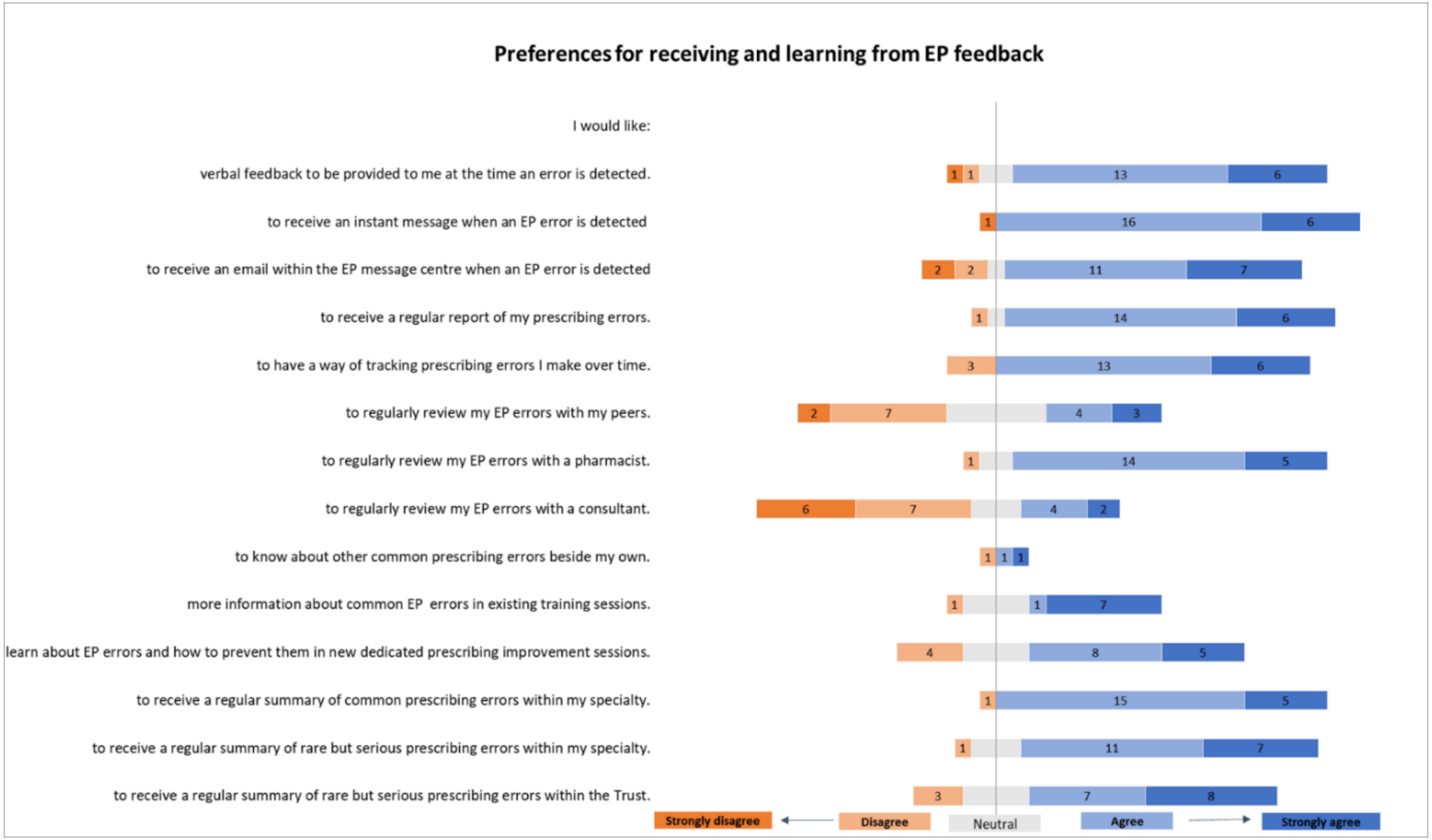

Figure 3. Survey results: preferences for receiving and learning from electronic prescribing (EP) feedback reported by Foundation doctors (numbers correspond with number of respondents, total 25 participants)

Appendix 1 Questionnaire template

Appendix 1:

Junior doctors' prescribing safety survey

Improving safety and effectiveness of electronic prescribing – how can training and learning from errors be improved?

Invitation: **All foundation year 1 and 2 doctors** at Imperial College Healthcare NHS Trust.

This questionnaire forms part of a study that aims to improve prescribing safety in hospitals with electronic prescribing systems. It centres on how the Trust can help make it easier to prescribe safely and effectively, particularly in the way in which electronic prescribing training and feedback about errors are currently provided. It also asks about your preferred methods for receiving feedback on your prescribing errors and learning from prescribing errors. Participation is voluntary, and all information will be treated confidentially. **If you have any questions, please contact** monsey.mcleod@nhs.net **on behalf of the Imperial Prescribing Feedback team.**

In the following questions, please select the answer that best represents your opinion by circling it. (**SD**= Strongly Disagree, **D**= Disagree, **N**= Neutral, **A**= Agree, **SA**= Strongly Agree)

A. YOUR PRESCRIBING AND FEEDBACK EXPERIENCE

I am confident in prescribing the correct and appropriate **non-intravenous** medications for patients via Cerner.		SD	D	N	A	SA
I am confident in prescribing the correct and appropriate **intravenous** medications for patients via Cerner.		SD	D	N	A	SA
I would prefer to use a paper drug chart.	Never used	SD	D	N	A	SA
I think I am always informed if I make a prescribing error.		SD	D	N	A	SA
It concerns me I may make repeated prescribing errors because I am not informed of them initially.		SD	D	N	A	SA
I think receiving formal feedback on prescribing errors makes it less likely that I make errors in the future.		SD	D	N	A	SA
Any feedback I receive is generally unscheduled/unplanned.		SD	D	N	A	SA

B. QUALITY IMPROVEMENT

I believe electronic prescribing has reduced the number of prescribing errors that result in actual patient harm.	Don't know	SD	D	N	A	SA
I believe monitoring and learning from prescribing errors is important.		SD	D	N	A	SA
I believe prescribers should receive feedback about their own prescribing errors.		SD	D	N	A	SA
I believe being aware of my own prescribing errors is important to improve my prescribing.		SD	D	N	A	SA

C. TRAINING

Overall, I am satisfied with electronic prescribing training I receive.		SD	D	N	A	SA
I am satisfied with the following: ePrescribing training provided at induction.	Not received	SD	D	N	A	SA
I am satisfied with the ePrescribing classroom training session provided by the Trust Information Communications and Technology (ICT) team.	Not received	SD	D	N	A	SA
I am satisfied with the e-learning prescribing training module.	Not received	SD	D	N	A	SA
I am satisfied with the specialty-specific prescribing training I receive.	Not received	SD	D	N	A	SA

For peer review only - http://bmjopen.bmj.com/site/about/guidelines.xhtml

Appendix 1 Questionnaire template

I am satisfied with the prescribing training provided as part of the postgraduate education sessions for F2/CMT (covers general prescribing safety including electronic prescribing tips).	Not received	SD	D	N	A	SA

D. RESOURCES

Overall, I am satisfied with the resources I can access to prescribe safely.		SD	D	N	A	SA
When relevant, I like to use the 'EPA quick reference guide' and crib sheets on the Source to help me prescribe.	Not used	SD	D	N	A	SA
In general, I mostly contact the pharmacy EPA team for technical prescribing queries relating to the Cerner system.	Not used	SD	D	N	A	SA
In general, I mostly ask **nurses** for help whilst prescribing (for technical Cerner-specific queries or medication-related queries).	Not used	SD	D	N	A	SA
In general, I mostly ask the **ward pharmacist** for help whilst prescribing (for technical Cerner-specific queries or medication-related queries).	Not used	SD	D	N	A	SA
In general, I mostly ask **my peers** for help whilst prescribing (for technical Cerner-specific queries or medication-related queries).	Not used	SD	D	N	A	SA
In general, I mostly ask a **CMT** for help whilst prescribing (for technical Cerner-specific queries or medication-related queries).	Not used	SD	D	N	A	SA
In general, I mostly ask the **consultant** for help whilst prescribing (for technical Cerner-specific queries or medication-related queries).	Not used	SD	D	N	A	SA
In general, I mostly ask nurses for help whilst prescribing **out-of-hours**	Not used	SD	D	N	A	SA
In general, I mostly ask the on-call pharmacist for help whilst prescribing **out-of-hours**	Not used	SD	D	N	A	SA
In general, I mostly ask my peers for help whilst prescribing **out-of-hours**	Not used	SD	D	N	A	SA
In general, I mostly ask a CMT for help whilst prescribing **out-of-hours**	Not used	SD	D	N	A	SA

E. PROSPECTIVE FEEDBACK (specific error) INCLUDING EXAMPLE SCENARIOS

I want to know about all **major** prescribing errors I make.	SD	D	N	A	SA
I want to know about **all** prescribing errors I make, however minor.	SD	D	N	A	SA
I **ONLY** want to know about prescribing errors I make that have the potential to cause patient harm.	SD	D	N	A	SA
I would like verbal feedback to be provided to me at the time the error is detected.	SD	D	N	A	SA
I would like to be able to receive an instant message when a prescribing error I've made has been detected.	SD	D	N	A	SA
I would like to be able to receive an email within the Cerner message centre when a prescribing error I've made has been detected.	SD	D	N	A	SA

I would like to receive feedback if I made this type of prescribing error:

An 82-year-old male patient with hospital acquired pneumonia was prescribed temocillin 2g IV BD and amoxicillin IV 1g TDS. He had a penicillin allergy with known anaphylaxis but his allergy status had not been completed on the electronic prescribing system. The error was detected and corrected before it reached the patient.	SD	D	N	A	SA
A 67-year-old female admitted for elective right hip replacement was started on prophylactic antibiotics at induction. Cefuroxime 1.5g IV TDS was prescribed post-op, intended for 24 hours but no stop date was prescribed. The patient received cefuroxime 1.5g IV TDS for two extra days before it was stopped. The patient has not reported any side effects from the prolonged course.	SD	D	N	A	SA
A 55-year-old male with type 2 diabetes and hypertension was admitted. One of their medications, ramipril 10mg OD, was not prescribed and the patient missed their dose for 3 days over the weekend. Their blood pressure had increased gradually and is now 190/110 mmHg.	SD	D	N	A	SA

For peer review only - http://bmjopen.bmj.com/site/about/guidelines.xhtml

Appendix 1 Questionnaire template

A 11-month old girl was prescribed amoxicillin 125mg/5ml suspension twice. One was for 2.5ml TDS for one week, and the other for 5ml TDS for one week. The error was corrected before it reached the patient.	SD D N A SA

F. RETROSPECTIVE FEEDBACK (error trends specific to my own prescribing)

I would like to receive a regular summary of my prescribing errors.	SD D N A SA
I would like to have a way of tracking prescribing errors I make over time.	SD D N A SA
I would like the opportunity to regularly review my prescribing errors with **my peers**.	SD D N A SA
I would like the opportunity to regularly review my prescribing errors with a **pharmacist**.	SD D N A SA
I would like the opportunity to regularly review my prescribing errors with a **consultant**.	SD D N A SA
Feedback (review) sessions about prescribing errors of relevance to my specialty would be most valuable on the **ward**, (e.g. at handover, as part of ward round).	SD D N A SA

G. AGGREGATED RETROSPECTIVE FEEDBACK (of prescribing errors within Imperial)

I would like to know about other common prescribing errors besides my own.	SD D N A SA
I would like more information about common prescribing errors in existing training sessions.	SD D N A SA
I would like to learn about prescribing errors and how to prevent them in new dedicated prescribing improvement sessions.	SD D N A SA
I would like to receive a regular summary of **common** prescribing errors within my specialty.	SD D N A SA
I would like to receive a regular summary of **rare but serious** prescribing errors within my **specialty**.	SD D N A SA
I would like to receive a regular summary of **rare but serious** prescribing errors within the **trust**.	SD D N A SA

Please answer the following questions.
If you had enough time, what three things would make it easier for you to prescribe safely and efficiently?

What are the main concerns regarding the current feedback you receive, if any, on prescribing errors?

Sex: F / M	Grade:	Medical school qualified from:

Please email monsey.mcleod@nhs.net on behalf of the Imperial Prescribing Feedback team for any of the following:

☐ I would like to receive a copy of the survey results
☐ I am happy to be contacted to clarify my answers above
☐ I am happy to be contacted about future improvement projects

Thank you for completing this questionnaire.
Please return to this to the Postgraduate Medical Education team at your site (directly or internal mail).

For peer review only - http://bmjopen.bmj.com/site/about/guidelines.xhtml

Appendix 2 Interview Topic Guide

Prescribing Safety Feedback Interview

Thank you for taking the time to participate this study. My name is XX and I am currently doing some research into feedback on prescribing errors within this trust.

Have you had a chance to look at the participant information sheet? Do you still have any questions? Are you happy for us to take notes? Are you happy to sign this consent form? No identifiable information is being recorded.

This interview is part of a larger study to explore the views of junior doctors and other stakeholders about providing feedback concerning prescribing errors. The interview focuses on how best to provide feedback to foundation year doctors to help make it easier to prescribe safely on the electronic system. This includes receiving feedback on prescribing errors as well as tips for how to prevent them. Participation is voluntary, and these interviews are completely anonymous.

Selection of prescribing error related feedback strategies

We know from a recent survey at this trust that FY1 and FY2 doctors do want to receive feedback about their own prescribing errors and also learn from errors elsewhere. In response to this, we are now looking to get your views on a range of different methods for receiving prescribing feedback.

PART 1

I will show you a few ways of providing feedback to support learning from prescribing errors

and would like your feedback on each strategy. [ACTION: give out documents – see below].

Questions to ask while they are looking at the documents:

1. Which of these feedback reports do you prefer? Please rank in order of preference.

- *[Wait until they have put them in order of preference, indicating which they think is*

best to worst (or if the same)]

2. Can you tell me the reasons for your answer?

3. What changes to this feedback document would make this more useful to you?

a. Prompts: What would you like to see changed about the feedback concerning

prescribing errors?

b. Prompts: Do you identify any problems with this feedback report? If you do,

which problems?

c. Prompts: What do you like about the presentation style?

74

d. Prompts: Is the information presented clear to you? Is the text big enough?

For peer review only

Appendix 2 Interview Topic Guide

Do you like the length of the feedback document or do you think we need to add/remove some information?

4. If you want to receive feedback, how often do you want to receive a document like these?

a. Prompts: Would you prefer to receive a feedback document weekly or monthly or other?

PART 2

Thank you, now I would like you to look at the lists below.

1. 1ST column - please tick the top 5 types of data you would like to receive feedback on and why.

2. 2nd column – please rank in order the denominators you would like to be used to determine the error rate. Why?

NUMERATOR – **please tick top 5**<br>Types of prescribing error (irrespective of actual or potential patient harm)	DENOMINATOR – **please rank (1 = most preferred info, 7 = least preferred info)**
☐ Allergy<br>☐ Omission of medication<br>☐ Unnecessary prescribing<br>☐ Clinical contraindications<br>☐ Dosing errors<br>☐ Formulation errors<br>☐ Wrong route errors<br>☐ Lack of clear directions for administration<br>☐ Technical errors (e.g. no max dose)<br>☐ Duration of treatment errors<br>☐ Miscellaneous<br>☐ Other (please state):	☐ No. of NEW medications prescribed (any time)<br>☐ No. of NEW medications prescribed 9am – 5pm<br>☐ No. of NEW medications prescribed out of hours<br>☐ No. of NEW medications prescribed (by day of week)<br>☐ No. of patients under the doctor's care (by consultant)<br>☐ No. of patients in which the doctor had prescribed at least one drug (irrespective of consultant/specialty/ward)<br>☐ Other (please state):

Did you receive feedback in the past via a different way than the methods discussed in this interview?

Is there anything else you would like to add?

Thank you for participating on this interview. Your contribution is very much appreciated

For peer review only

Appendix 2 Interview Topic Guide

Example 1

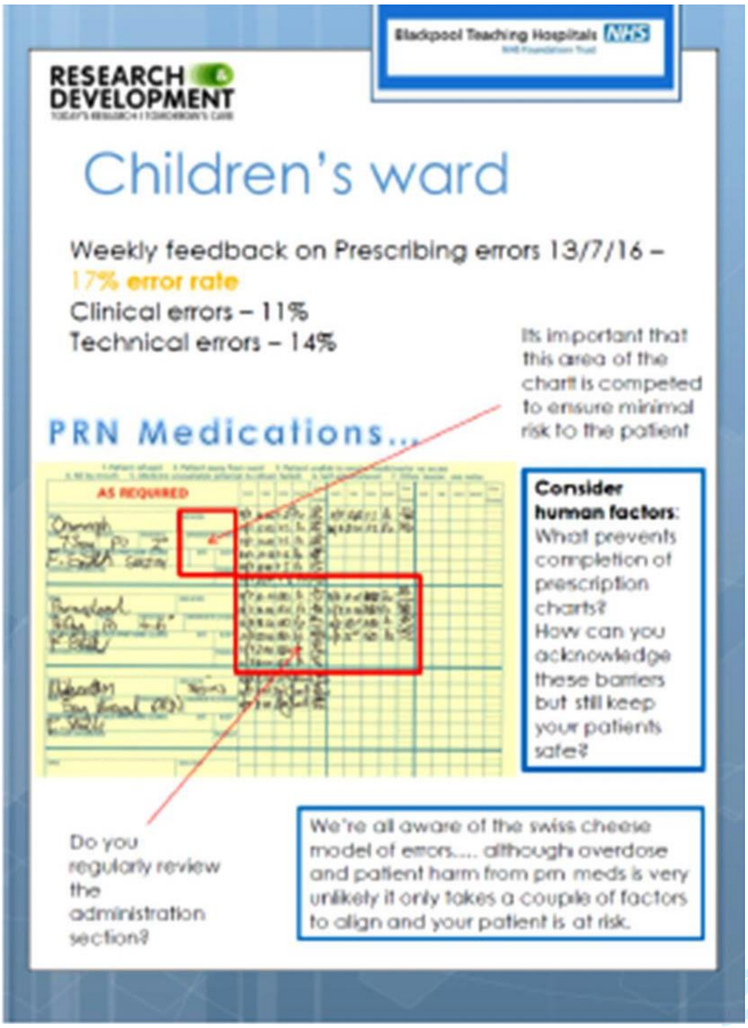

Appendix 2 Interview Topic Guide

Example 2

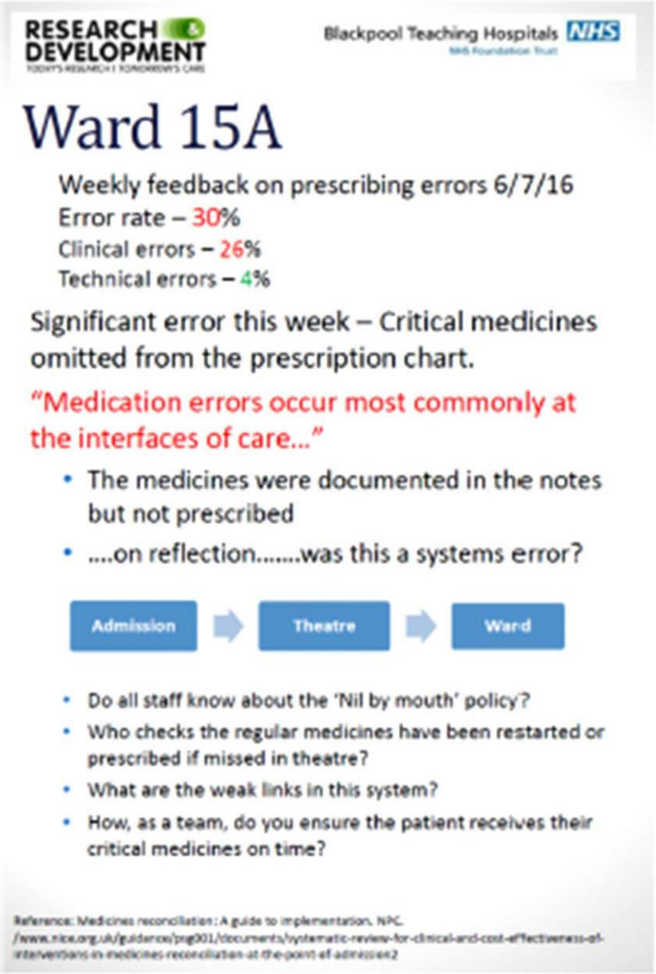

Appendix 2 Interview Topic Guide

Example 3

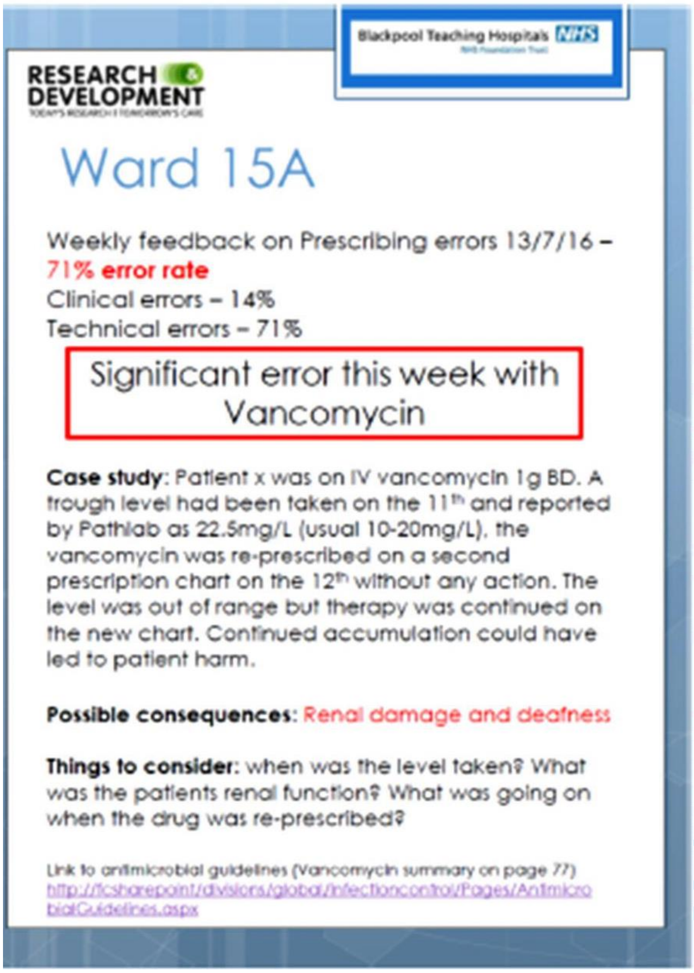

Appendix 2 Interview Topic Guide

Example 4

Western Sussex Hospitals Standard Process for Routine Ward Round
Considerative Checklist for Complete Patient Review

The purpose of this process is to ensure that the team "does it all" for all the patients during the round
Make one member of the team the "Safety Checker" who uses this checklist before leaving each patient.
The checker must highlight anything omitted, speak up and get it done!
Key = [yellow box] these sections must be checked in all patients, tick white boxes only when indicated

Date	Checker's Name	Checker's Status	Signed	Clinical Team	Type of Round
…/…./2015					**Routine**
Start time	Finish time	Number of Doctors	**Total number of patients**		
			**New**	**Review**	**No of wards**

Aspect of Care										
**Patient Initials**										
**Bed number**										
**Preparatory Discussions**	**Preparation Before Going to the Bedside**									
**Review Diagnoses**										
**Checked New Results**										
Clinical Thinking										
**Clinical Frailty?**										
**Report from Nurse?**										
**Consultation**	**Bedside Patient Consultation**									
**Nurse present?**										
**Hand hygiene?**										
**Introductions?**										
**Wristband check?**										
**Ask and Listen?**										
**Charts**	**Check All Relevant Bedside Charts**									
**Write the NEWS Score**										
**Drugs Chart checked?**										
On iv Fluid? Review chart										
Diabetic? Glucose levels										
**Planning**	**Decide on blood tests, radiology, Discuss Discharge Date, Ceiling of Care**									
Agree future tests?										
EDD discussed?										
MRFD?										
**7 Point Safety Check**	**7 Point Safety Check to Reduce Avoidable Harm**									
Pain or discomfort										
Eating and Drinking										
Bowel Function										
Urine / catheter?										
Cannula and iv lines										
Pressure area care										
Change VTE Rx?										
**All 7 points checked?**										
**Documentation**	**Consultant Inspects and Counter Signs Today's Notes**									
**Notes signed by Consultant?**										
**Summed Up to Patient?**										
**Reported Plan to Nurse?**										

© Dr G Caldwell January 2011

H:_1_Gordon_Assessment_and_Feedback_1Ward Round Pack_SOP Routine Ward Round 21150217.docx

Appendix 2 Interview Topic Guide

Example 5

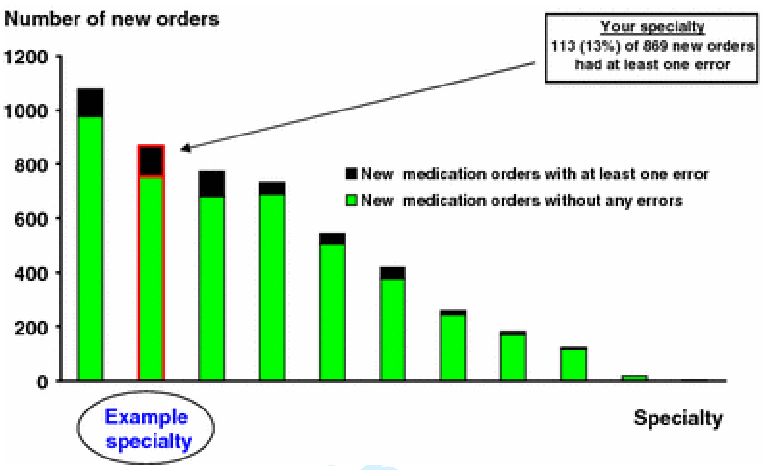

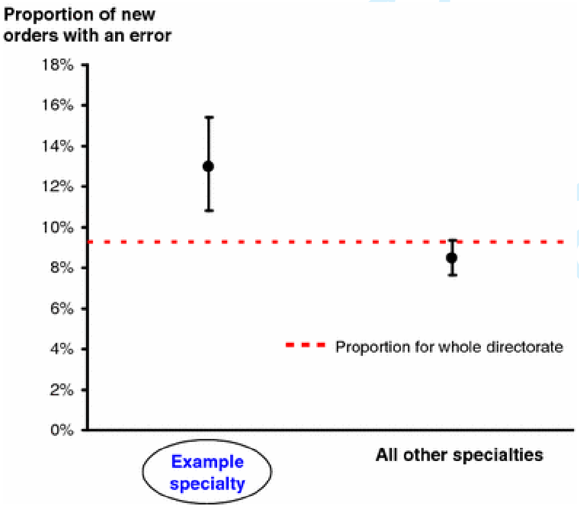

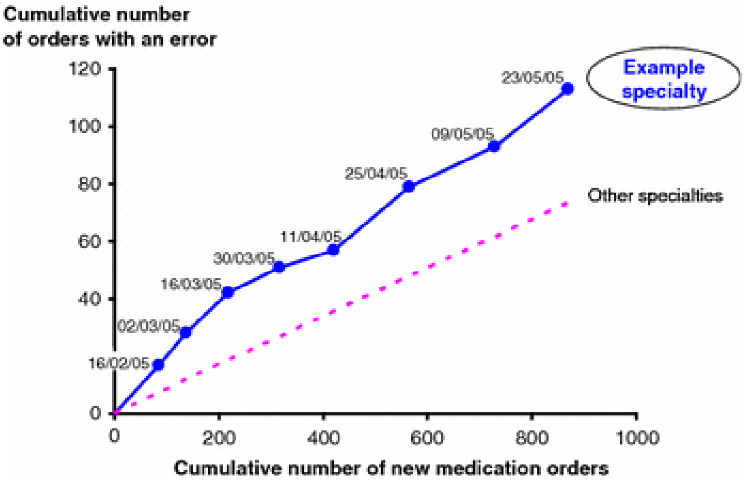

Appendix 2 Interview Topic Guide

Example 6

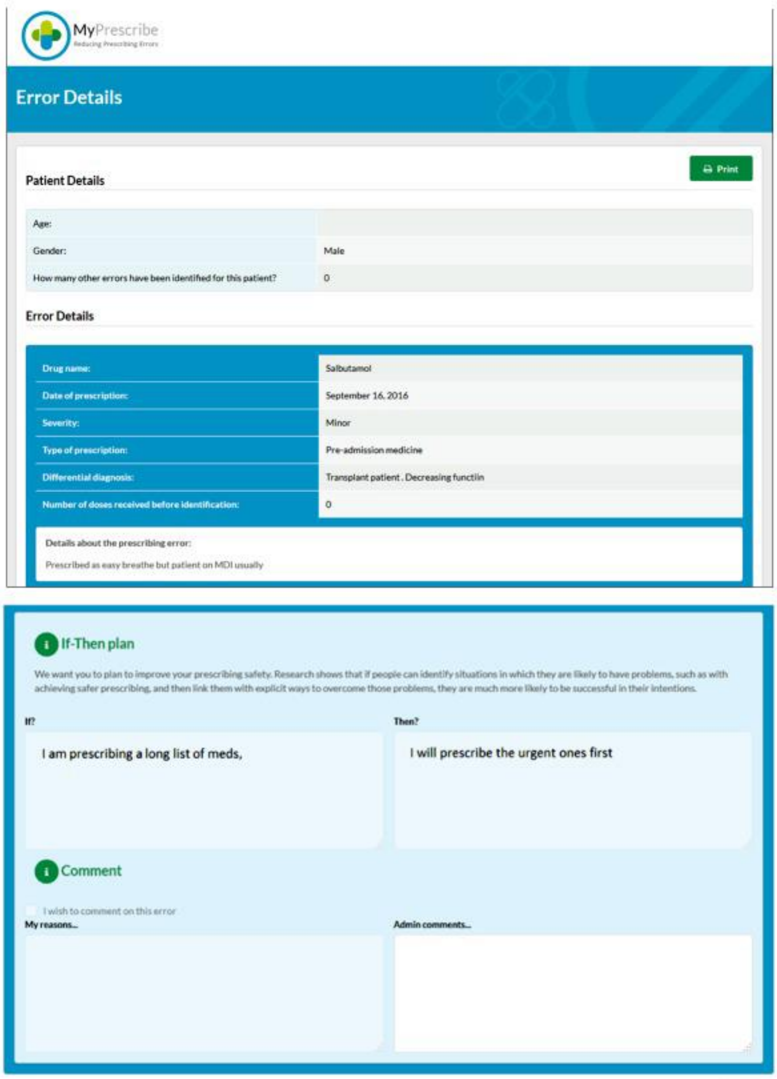

Appendix 3 Thematic Framework

Theme	Sub-theme	Codes
**Nature of individualised feedback data**	*Timing of feedback*	Timely feedback especially for significant errors
		Weekly feedback appropriate for minor or general errors
		Timely feedback really important
		Timely feedback to optimise recall and context – weekly or fortnightly
		Weekly feedback
		Timely personal feedback e.g. weekly
		Team every 2 weeks
		Timely feedback – weekly or fortnightly
	Characteristics of feedback	Feedback not helpful if too generic
		Tailoring the feedback e.g. selecting the most common error or significant error in that report
		Positive as well as negative feedback
		Keep feedback brief
		Short and personalised report
		Feedback needs to be specific
		Non-judgemental
		Qualitative feedback better than quantitative
		Needs to be motivational
	Content format	Combination of graphs and text
		Combination of content:
		Text poster - weekly feedback error rate + picture of example drug chart
		Text poster – weekly error rate + tips/questions for improvement
		Overview of prescribing error rate but less frequently
		Mixed feelings about error rates
		Poster with weekly error rate and example drug chart highlighting mistake
		Useful formats:
		Text poster – weekly error rate + tips/questions for improvement

For peer review only

Appendix 3 Thematic Framework

		Text poster – significant error highlighted with case study
		Basic qualitative info would be helpful for context
**Context of feedback**	*Relationship with feedback provider*	Feedback in person from the person correcting the mistake is more helpful
		Not received formal feedback before
		Mistakes corrected by pharmacist and feedback usually casual
		Specialised pharmacist can provide context
		From anonymised source
	Importance of error	Feedback on significant errors – actual or potential harm to patients
		Prescribing tips sometimes helpful but not always significant
		Recognised importance of major and minor errors for safe patient care
		Minor errors indicate 'laziness'
		Feedback focussed on serious errors
		Especially for significant error
		Significant errors by others would be useful for learning
	Time to focus on feedback	Workload pressure – doctors not good at focussing on emails
		Implementing change is difficult – better to start at the beginning of a cohort
		Not always time to read emails
		Workload pressures
**Learning from feedback**	*Data validity*	Participant unclear about how data will be captured
		How to collect the data
		Equivocal about graphs – comparisons between teams and specialties may not be valid, minor and serious errors may not be separated
		Error rate on its own is not helpful
	Benchmarking with peers	Benchmarking against peers could be helpful but numbers might be too small

For peer review only

Appendix 3 Thematic Framework

		The person who made the error might feel bad though
	Generic case studies/examples	Learning from others' mistakes especially as Foundation doctors rotate specialties
		Learning from case study helpful
		Case studies helpful
		Learning from case study
	Teamworking culture	Personalised feedback but also in context of team prescribing culture
		Role modelling or bad habits from senior doctors
		Team hierarchy
		Feedback to team
		Team feedback and culture very important
		Personal relationships help with feedback e.g. ward or team pharmacist
		But if there is little existing relationship, better to receive a document
**EP-specific feedback**	*EP errors*	Electronic prescribing hard to adjust to from paper system
		There are some advantages to the paper charts not easily transferred to EP system e.g. suspending a drug, alternate day dosing, stopping medicines, holding a medication pending review
		EP errors e.g. meds being administered 'now' rather than pre-set times
	Other	Preparedness – national prescribing exam too easy

For peer review only
